# Palladium(II) Complexes with Chloro-Substituted Salicyl Schiff Bases: Exploring Multimodal Anticancer Mechanisms and Catalase Inhibition

**DOI:** 10.3390/molecules31081370

**Published:** 2026-04-21

**Authors:** Jovana S. Dragojević, Žiko Milanović, Kristina Milisavljević, Milena Milutinović, Safija Herenda, Edhem Hasković, Nenad Vanis, Vera M. Divac, Marina D. Kostić

**Affiliations:** 1 Department of Chemistry, Faculty of Science, University of Kragujevac, Radoja Domanovića 12, 34000 Kragujevac, Serbia; jovana.marjanovic@pmf.kg.ac.rs (J.S.D.); vera.divac@pmf.kg.ac.rs (V.M.D.); 2 Department of Science, Institute for Information Technologies, University of Kragujevac, Liceja Kneževine Srbije 1A, 34000 Kragujevac, Serbia; ziko.milanovic@uni.kg.ac.rs (Ž.M.); kristina.milisavljevic@uni.kg.ac.rs (K.M.); 3 Department of Biology and Ecology, Faculty of Science, University of Kragujevac, Radoja Domanovića 12, 34000 Kragujevac, Serbia; milena.milutinovic@pmf.kg.ac.rs; 4 Department of Chemistry, Faculty of Science, University of Sarajevo, 71000 Sarajevo, Bosnia and Herzegovina; safija@pmf.unsa.ba; 5 Department of Biology, Faculty of Science, University of Sarajevo, 71000 Sarajevo, Bosnia and Herzegovina; e.haskovic@pmf.unsa.ba; 6 ASA Hospital, Džemala Bijedica 127, 71000 Sarajevo, Bosnia and Herzegovina; nenad.vanis@ssst.edu.ba

**Keywords:** palladium, Schiff base, antitumor activity, catalase inhibition, molecular docking

## Abstract

The search for new anticancer agents with improved efficacy and reduced toxicity has intensified interest in metal-based compounds. In this study, two novel palladium(II) complexes, synthesized from Schiff base ligands derived from 5-chloro-salicylaldehyde and *p*-hydroxybenzylamine or tyramine, were chemically characterized and biologically evaluated. Both complexes exhibited significant cytotoxic activity against the MCF-7 breast cancer cell line in a dose- and time-dependent manner, with **Pd2** showing slightly higher potency. Morphological analysis of treated cells indicated that apoptosis is the predominant mechanism of cell death. To gain deeper insight into the potential mechanisms underlying the observed anticancer activity, several biologically relevant targets were investigated. Enzyme kinetics revealed that the complexes act as uncompetitive inhibitors of liver catalase, suggesting a possible role in the induction of oxidative stress. Fluorescence studies demonstrated that **Pd2** interacts with CT-DNA through combined intercalative and minor groove binding modes and exhibits significant binding affinity toward human serum albumin, predominantly at Sudlow’s site I. Molecular docking analysis further supported favorable interactions with catalase, estrogen receptor α, and B-form DNA, providing structural insight into the experimentally observed biological effects. Overall, the study explores multiple potential mechanisms of anticancer action, underscoring the promising therapeutic potential of these palladium(II) complexes, while antitumor activity has been initially assessed using a MCF-7 cell line as a preliminary model.

## 1. Introduction

The design and synthesis of novel antitumor drugs with improved selectivity and low toxicity is one of the major goals of modern science. Metallo-drugs deserve special attention in this area, mainly due to the unique chemical properties reflected in a variety of coordination modes and redox properties [1,2]. Platinum-based agents have long played a central role in cancer chemotherapy, with Cisplatin representing the prototype of this class. Although initially synthesized as early as 1844 by Michele Peyrone, its biological activity remained unrecognized until Barnett Rosenberg reported its antitumor properties in 1965. This discovery occurred serendipitously during experiments investigating the effects of electric currents on the growth of Escherichia coli, where inhibition of cell division was traced back to the formation of cisplatin from platinum electrodes. Subsequent in vivo studies led to clinical trials initiated in the early 1970s, culminating in the approval of cisplatin in 1978 for the treatment of testicular and ovarian cancers. Since then, its clinical use has expanded to a wide range of malignancies, including bladder, lung, cervical, and head and neck cancers. Although the platinum compounds have been applied as adjuvant therapy in the treatment of a large spectrum of cancers [3,4,5], their side effects and drug resistance are imposing the need for novel antitumor solutions from the metallo-drug area [6]. In the search for improved anticancer agents, transition metal complexes have attracted considerable attention, particularly those based on palladium [7,8,9,10,11]. Palladium complexes share notable chemical similarities with their platinum counterparts, as both metals belong to the platinum group elements and commonly exist in comparable oxidation states. Structural parallels are reflected in similar bond lengths and coordination geometries. However, palladium compounds exhibit significantly faster ligand exchange kinetics, making them substantially more reactive. This increased reactivity necessitates stabilization through appropriate ligand design, often involving chelating systems. The differences in behavior between platinum and palladium can be attributed to their electronic configurations, which influence bond strength, ionization potential, and overall stability of the resulting complexes. Palladium has found its place in medicine, such as ^103^Pd radioactive plaque radiotherapy of intraocular melanoma [12] or as alloys for dental applications [13]. Due to the similar electronic configuration and significant structural resemblance to platinum, palladium has inspired the development of Pd(II) complexes as more effective and less toxic alternatives to the platinum-based antitumor agents [14,15,16,17]. All these studies have helped in the deeper understanding of the underlying relationships between the complex structures and exhibited activities. Although platinum-based drugs remain widely used in clinical practice, their limitations—including poor water solubility, significant side effects, and resistance development—have driven ongoing efforts to identify alternative metal-based therapeutics. In this context, palladium complexes have emerged as promising candidates, motivating further investigation into their chemical properties and biological activity. The key step in the design of metallo-drugs suitable to serve as antitumor agents is the proper choice of organic ligand, since the delicate changes in the ligand structure and introduction of adequate substituents can provide desired pharmacological and physicochemical properties to metal complexes. Schiff base ligands received enormous scientific attention due to the versatile coordination modes and wide range of physicochemical properties [18,19,20,21,22,23,24]. Among them, particularly those bearing a salicylic structural motif have been studied intensively during the last years [25,26,27,28,29]. The heightened interest arises from their structural flexibility, ease of functional modification, and strong propensity to form stable metal complexes that often exhibit enhanced biological activity relative to the free ligands.

One of the potential mechanisms underlying the antitumor activity of drugs is catalase inhibition, which leads to elevated intracellular oxidative stress, increased susceptibility of cancer cells to damage, and consequently enhanced antitumor efficacy. Palladium complexes capable of inhibiting catalase have attracted increasing attention due to their potential biological and therapeutic relevance [30,31,32,33]. Catalase is a key antioxidant enzyme responsible for the decomposition of hydrogen peroxide, and its inhibition can lead to elevated intracellular reactive oxygen species (ROS) levels. This catalase inhibition is biologically significant because increased ROS levels can promote oxidative stress in cancer cells, which are often more vulnerable to redox imbalance than normal cells. As a result, palladium complexes that inhibit catalase may enhance antitumor activity by triggering apoptosis, disrupting cellular metabolism, and sensitizing tumor cells to oxidative damage. These properties position catalase-inhibiting palladium complexes as promising candidates for the development of novel metal-based anticancer agents.

In light of the above and our previous experience in this field [26,34,35], this study reports the synthesis, structural characterization, and in vitro cytotoxic activity against MCF-7 cells of two novel palladium(II) complexes, **Pd1** and **Pd2**, derived from Schiff bases **1** and **2** formed by condensation of 5-chloro-substituted salicylaldehyde with *p*-hydroxybenzylamine or tyramine. The incorporation of a chloro substituent was expected to enhance lipophilicity and promote hydrophobic interactions with potential biological targets, thereby improving the biological activity of the ligands [36]. In addition, the structural difference between *p*-hydroxybenzylamine and tyramine, consisting of the presence or absence of a methylene linkage between the aromatic ring and the amino group, may influence molecular flexibility, binding orientation, and overall biological effectiveness. To gain deeper insight into the potential anticancer mechanisms of these complexes, several biologically relevant interactions were investigated, including the inhibitory effect on catalase at different complex concentrations, as well as interactions with DNA and human serum albumin. Furthermore, considering the estrogen-dependent nature of MCF-7 cells, the potential interaction with estrogen receptor α was also examined. Complementary molecular docking studies were also conducted to provide structural insight into the observed biological activity and to explore possible interactions with catalase, estrogen receptor α, and DNA. Overall, the multimodal anticancer mechanisms of the synthesized palladium(II) complexes were investigated, including cytotoxicity assessment, catalase inhibition, DNA interaction studies, and molecular docking analyses to explore biomolecular binding behavior. Experimental biological evaluation was performed using the MCF7 cell line as an initial model system.

## 2. Results and Discussion

### 2.1. Synthesis of Complexes ***Pd1*** and ***Pd2***

**Scheme 1:**
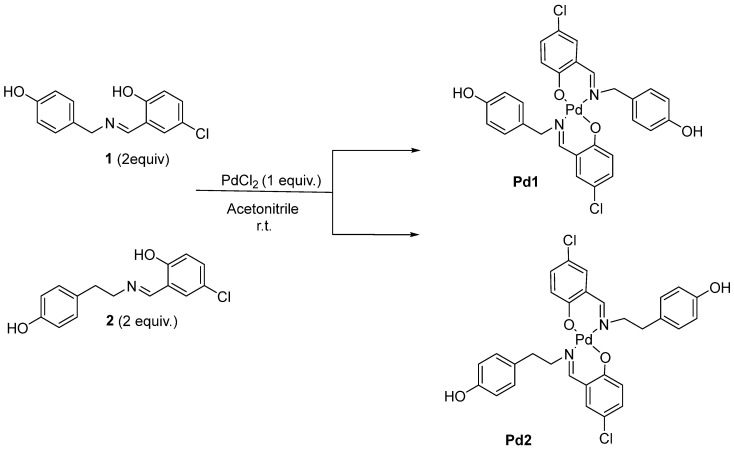
Synthesis of palladium complexes **Pd1** and **Pd2**.

Schiff base ligands have been synthetized in one-pot procedure consisting of absolute ethanol-driven reaction between equimolar amounts of 5-chlorosalicylic aldehyde and para hydroxy benzyl amine/or tyramine. Of the ligands studied, Schiff base **2** is known from the literature [37], whereas compound **1** is a novel compound, hitherto unreported. The general procedure for the synthesis of all compounds and their spectral data is described in the Experimental part, and all spectra are given in the Supplementary Material. Further, these ligand compounds have been utilized for the preparation of palladium(II) complex species, as depicted in Scheme 1. The reaction procedure encompasses the reaction between the corresponding ligand compound (**1** or **2**) with palladium chloride in a molar ratio of 2:1, in acetonitrile, at room temperature. The obtained yellow powders were characterized using NMR and IR spectroscopy, mass, and elemental analysis.

The synthesized complex involves a Schiff base ligand acting as a bidentate N,O-donor, coordinating to the Pd(II) center through the imine nitrogen and phenolic oxygen atoms. With two such ligands bound to the metal center, a square-planar coordination environment is formed, which is typical for Pd(II) complexes. The preference for the trans configuration is consistent with previously reported Pd(II) complexes containing similar N,O-bidentate Schiff base ligands, where steric and electronic factors favor the trans arrangement over the cis one [38]. The obtained complexes exhibit a 1:2 metal-to-ligand stoichiometry, as confirmed by mass spectrometric analysis. This can be attributed to the preferred square-planar coordination geometry of palladium(II), which allows coordination of two ligand molecules. Under the applied reaction conditions, the equilibrium is shifted toward the formation of the bis-ligand complex, resulting in the isolation of the PdL_2_ species.

### 2.2. Structural Characterization of Complex Compounds ***Pd1*** and ***Pd2***

In the FT-IR spectrum, the characteristic imine (C=N) stretching band, originally observed around 1620–1650 cm^−1^ in the free ligand **1** and **2** (1644 and 1625, respectively), exhibited a noticeable shift to lower wavenumbers (1625 and 1621), indicating coordination of the nitrogen atom to the palladium center (Table 1). Similarly, the phenolic –OH stretching band, typically appearing near 3400–3450 cm^−1^ in the ligand compounds, was either significantly broadened or shifted, suggesting involvement of the hydroxyl group in metal binding.

The ^1^H NMR spectra provided clear evidence of coordination, as the –OH proton signals at 13.57 ppm (Schiff base **1**) and 13.64 ppm (Schiff base **2**) disappeared due to deprotonation and metal binding, while the imine proton signals at 8.63 ppm and 8.44 ppm (for **1** and **2**, respectively) were slightly deshielded to 7.99 and 7.89 ppm in complexes, confirming involvement of both the hydroxyl oxygen and imine nitrogen in complex formation. The characteristic chemical shifts summarized in Table 1 are also clearly observed in the representative example of the NMR spectra presented in Figures S5 and S9 in the Supplementary Materials.

The mass spectrometric analysis of the palladium complex provided further insight into its stoichiometry. The molecular ion peaks [M + H]^+^ from 629.0054 (for **Pd1**) and 657.0373 (for **Pd2**) corresponded to a molecular weight consistent with a 1:2 metal-to-ligand ratio, indicating that two Schiff base ligands are coordinated to a single palladium center (Supplementary Materials, Figures S8 and S12). Fragmentation patterns further supported this assignment, showing characteristic losses corresponding to ligand moieties while preserving the integrity of the coordinated complex. These results confirm that the palladium ion is chelated by two ligand molecules, forming a stable bidentate complex.

The molar conductivities of complexes **Pd1** and **Pd2** were determined in DMSO and found to belong to the values of 15.2 and 16.9 S cm^2^ mol^−1^, respectively. These low conductivity values are consistent with literature reports for palladium complexes coordinated by two ligand molecules, indicating a neutral nature and nonelectrolytic behavior in solution [39].

### 2.3. Assessment of Cytotoxic Activity

#### 2.3.1. MTT Assay on MCF-7 Cell Line

**Figure 1:**
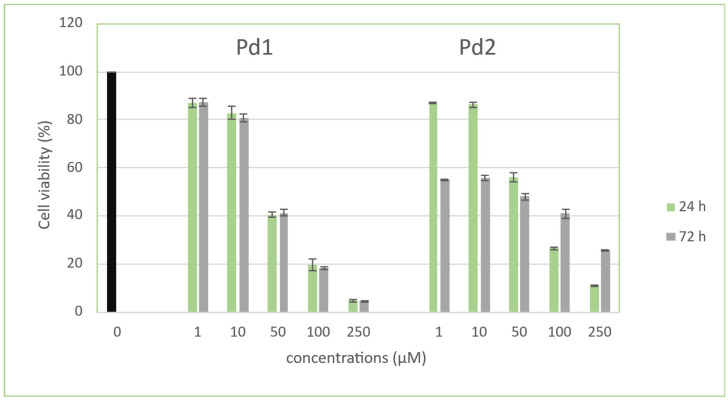
The MCF-7 cell viability in 24 and 72 h treatments with Pd complexes.

Using the MTT assay, the MCF-7 cell viability was initially determined and presented in Figure 1. The Pd complexes induced a significant (*p* < 0.05 in all applied doses compared to control) dose-dependent decrease in MCF-7 cell viability.

Based on observed viabilities, the IC_50_ values (concentration that induce 50% of cell death) were calculated and presented in Table 2. It is a commonly used value as a parameter for cytotoxicity in preclinical testing. These values for Pd complexes firstly indicate their pronounced cytotoxicity on MCF-7 cells. Time-dependent effects for both investigated complexes are also important, suggesting that cells do not recover after prolonged exposure. After 24 h, **Pd2** demonstrated higher cell viability (63.2 ± 1.5%) compared to **Pd1** (48.6 ± 1.6%), indicating a lower initial cytotoxic effect and better short-term biocompatibility. This suggests that **Pd2** is less aggressive toward the tested cells at earlier exposure times.

However, after 72 h, a different trend was observed. Cell viability decreased for both complexes, with **Pd2** showing a more pronounced reduction (40.7 ± 1.0%) compared to **Pd1** (46.4 ± 1.7%). This indicates that **Pd2** exhibits a stronger time-dependent cytotoxic effect, leading to reduced cell survival over prolonged exposure.

Overall, the results suggest that **Pd2** has a more favorable short-term profile in terms of cell viability, while **Pd1** maintains more stable cell viability over time. These findings indicate that the biological effects of Pd complexes are strongly dependent on exposure duration, highlighting the importance of time-dependent assessment when evaluating their cytotoxic potential.

#### 2.3.2. Analysis of Cell Morphology

**Figure 2:**
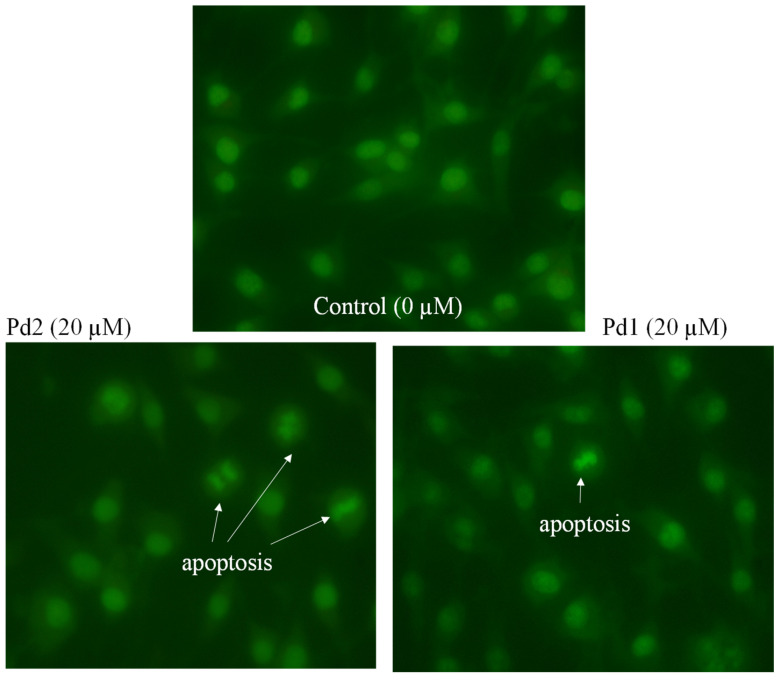
Morphological changes in MCF-7 cells occur under the influence of Pd complexes.

After observing noticeable cytotoxicity of the investigated Pd complexes, analysis of cell morphology was conducted to determine the predominant type of induced cell death. MTT assay gives information about cell proliferation and viability, but the following observation provides insight into the mechanism by which cytotoxicity is induced. This is crucial to ensure that no necrotic effects occur. The morphological changes observed in the treated samples included loss of adhesion and detachment from the culture plate, alterations in nuclear morphology such as chromatin condensation and nuclear fragmentation, and a stronger green, fluorescent signal (Figure 2). In addition, membrane blebbing and formation of apoptotic bodies were evident, all of which are characteristic features of apoptosis. These observations indicate that apoptosis is the primary mechanism of cell death induced by both Pd complexes. Necrotic events were rare and occurred only sporadically.

An important aspect of the obtained results is the mechanism of cell death induced by the investigated compounds. Experimental observations indicate that the cytotoxic effect is primarily associated with apoptosis rather than necrosis. Apoptosis represents a regulated and controlled form of programmed cell death, which is generally considered more desirable in anticancer therapy because it does not trigger inflammatory responses in surrounding tissues. In contrast, necrosis is an uncontrolled process that often leads to inflammation and damage to neighboring cells [42].

Therefore, the ability of the investigated palladium complexes to induce apoptotic cell death represents a particularly significant finding. This mechanism suggests a more selective and therapeutically favorable mode of action, which may reduce undesirable side effects commonly associated with many anticancer agents. Overall, the combination of very low IC_50_ values and the induction of apoptosis highlights the strong potential of these palladium complexes as promising candidates for further investigation in anticancer drug development.

#### 2.3.3. Influence of Structural Variation and Lipophilicity on Biological Activity

To examine the effect of structural variations—specifically, the single –CH_2_– difference between **Pd1** and **Pd2**, as well as a chloro-free model of **Pd1**—lipophilicity parameters (Log *P*_o/w_) were predicted using the WebADMET platform. Multiple computational models implemented within this tool (iLOGP, XLOGP3, WLOGP, MLOGP, and SILICOS-IT) were applied, and the reported consensus Log P values were used for discussion (Table 3), as they provide a more reliable assessment by combining different predictive approaches.

The lipophilicity of the investigated systems shows a clear and systematic trend across the series: **Pd1** (chloro-free model structure) < **Pd1** < **Pd2**, with consensus Log *P*_o/w_ values of 2.79, 3.74, and 4.18, respectively. This progression reflects the cumulative impact of structural modifications on the overall hydrophobic character of the complexes.

The comparison between **Pd1** (chloro-free model) and **Pd1** highlights the significant contribution of chloro substituents to lipophilicity. The increase in Log P (Δ ≈ 0.95) upon introduction of chloro groups indicates their strong electron-withdrawing and hydrophobic nature, which enhances the affinity of the complex for lipid environments.

A further increase in lipophilicity is observed from **Pd1** to **Pd2** (ΔLog P ≈ 0.44), despite the seemingly minor structural difference in a single –CH_2_– group. This result demonstrates that even small structural modifications can measurably influence physicochemical properties. The additional –CH_2_– group increases the hydrophobic surface area and molecular flexibility, contributing to higher lipophilicity.

From a biological perspective, this gradual increase in lipophilicity is highly relevant. Enhanced lipophilicity is generally associated with improved membrane permeability and more efficient cellular uptake. Therefore, the higher biological activity observed for **Pd2** compared to **Pd1** can be rationalized by its increased lipophilicity. Similarly, the significantly lower lipophilicity of the chloro-free model suggests reduced membrane affinity, supporting the importance of chloro substituents in modulating biological performance.

Overall, these results demonstrate that both the presence of chloro substituents and subtle structural variations, such as the introduction of a –CH_2_– group, play a crucial role in tuning lipophilicity and, consequently, the biological activity of Pd(II) complexes.

### 2.4. Inhibition of Liver Catalase by ***Pd1*** and ***Pd2*** Complexes

**Figure 3:**
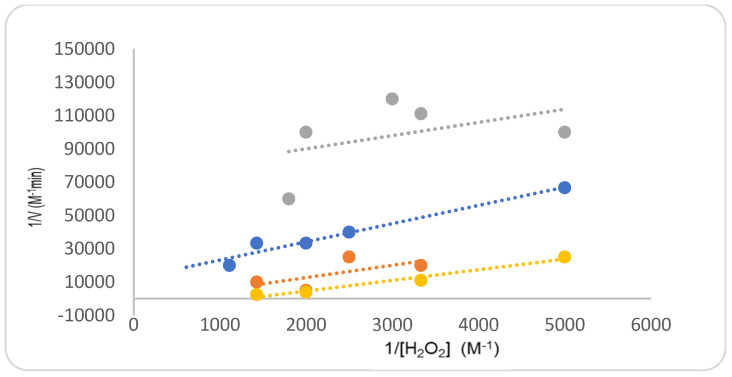
Lineweaver–Burk plot for determining Vmax and Km in the absence and presence of different concentrations of a Pd complex (**Pd1**): 0 mg/mL, 0.07 mg/mL, 0.11 mg/mL, 0.25 mg/mL.

**Figure 4:**
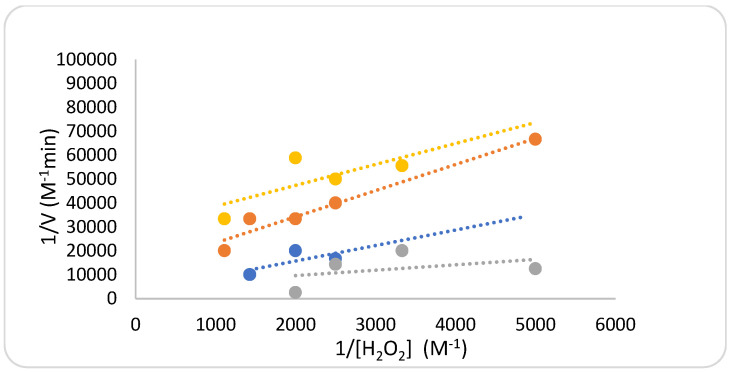
Lineweaver–Burk plot for determining Vmax and Km in the absence and presence of different concentrations of a Pd complex (**Pd2**): 0 mg/mL, 0.07 mg/mL, 0.11 mg/mL, 0.25 mg/mL.

In addition to the evaluation of cytotoxic activity, the inhibition of catalase was also investigated in order to obtain deeper insight into the possible mechanisms responsible for the observed antiproliferative effects. Catalase is a key antioxidant enzyme involved in the decomposition of hydrogen peroxide and the regulation of intracellular reactive oxygen species (ROS) [43]. Alterations in catalase activity can significantly affect the redox balance within cancer cells, potentially leading to oxidative stress and the activation of apoptotic pathways. Therefore, studying the type of catalase inhibition provides valuable information about the possible biochemical interactions of the investigated palladium complexes and may help to explain, at least in part, the pronounced cytotoxic effects observed in MCF-7 cells. To investigate the type of inhibitory effect of different concentrations of Pd complexes on enzyme activity over a range of hydrogen peroxide substrate concentrations (1.5–7.6 mM), the Michaelis–Menten kinetic model was employed. The Lineweaver–Burk plots (Figure 3 and Figure 4) indicate an uncompetitive type of enzyme inhibition, as evidenced by the presence of parallel lines. Inhibitors of this type require the prior formation of the enzyme–substrate (ES) complex for binding and inhibition. Consequently, such inhibitors affect catalytic steps that occur after the initial noncovalent binding of the substrate to the enzyme’s active site.

The binding of the Pd complex to the ES complex proceeds according to the following mechanism. In the first step, the substrate binds to the active site of the enzyme, followed by coordination of the Pd complex to the ES complex, resulting in the formation of an enzyme–substrate–palladium complex. Palladium (Pd) in these complexes typically exists in the +2 or 0 oxidation states and is coordinated by ligands. Pd complexes are known for their ability to coordinate with donor atoms (N, S, and O) in proteins, thereby interfering with the enzyme’s active site or inducing conformational changes in the protein structure. Palladium complexes are being investigated as antitumor agents due to their ability to deactivate enzymes such as catalase, leading to increased oxidative stress in tumor cells. However, inhibition of catalase may also result in toxicity, as accumulation of H_2_O_2_ can cause cellular damage.

Using the Lineweaver–Burk Equation (1), the kinetic parameters, i.e., the constants K_m_ and V_max_, were calculated for the corresponding concentrations of the Pd complex:

(1) $$\frac{1}{v_{0}}=\frac{K_{m}}{V_{max}}\frac{1}{\left[S\right]}+\frac{1}{V_{max}}\left(1+\frac{\left[I\right]}{K_{i}}\right)$$

The values obtained for V_max_ and K_m_ change because this is an uncompetitive type of inhibition. For the uninhibited reaction, the value of V_max_ is 0.81 × 10^−4^ while K_m_ is 0.88 mM. The K_m_ values in the presence of the Pd complex **Pd1** at different concentrations decrease from 2.37 mM to 0.24 mM, indicating the binding affinity of the Pd complex to the enzyme–substrate complex. The V_max_ values also decrease (3.66, 0.81, 0.34 × 10^−4^ Mmin^−1^) for the same complex and the concentration range of 0.07, 0.11, and 0.25 mg/mL.

At a fixed substrate concentration of 0.01 mM, the palladium complex **Pd1** shows 54.42% inhibition at a complex concentration of 0.11 mg/mL. In a comparative study, the IC_50_ value of the Pd(II) complex is very close to those of traditional AChE inhibitors (tacrine, rivastigmine, and galantamine) [44,45,46].

The K_m_ values in the presence of the Pd complex **Pd2** at different concentrations decrease to 3.38, 0.78, and 0.10 mM, as do the maximum reaction rates (4.56, 1.23, and 0.13 × 10^−4^ M min^−1^). When comparing the Km values of the complexes, it can be seen that the palladium complex **Pd2** has a stronger binding affinity for catalase. For the same substrate concentration and a fixed complex concentration, the IC_50_ value is 32.20%.

The cytotoxic effects of the investigated Pd complexes are closely associated with their inhibition of catalase. Both **Pd1** and **Pd2** act as uncompetitive inhibitors, binding preferentially to the enzyme–substrate complex and reducing Vmax and Km values. **Pd2** exhibits slightly stronger binding affinity than **Pd1**, consistent with its lower IC_50_ values. Morphological analysis of treated cells revealed hallmarks of apoptosis, including chromatin condensation, nuclear fragmentation, membrane blebbing, and formation of apoptotic bodies, while necrotic events were minimal. These findings suggest that catalase inhibition by the Pd complexes may lead to increased intracellular H_2_O_2_ levels, triggering regulated apoptotic cell death. Although direct ROS measurements were not performed, the combination of enzymatic inhibition and apoptosis strongly supports an oxidative stress-mediated mechanism, highlighting the potential of these Pd complexes as selective anticancer agents.

### 2.5. Biomolecular Interactions: DNA and HSA Binding Studies

#### 2.5.1. DNA Binding Studies

Numerous metal-based anticancer compounds have DNA as their primary biological target. Therefore, understanding the binding behavior of various transition metal ion complexes is of great importance. In general, these complexes can interact with DNA through two main types of binding: covalent and non-covalent. Covalent interactions involve the substitution of a labile ligand in the metal complex by a nitrogen atom from a DNA base, whereas non-covalent interactions include intercalation, electrostatic attraction, and groove binding. Fluorescence quenching is one of the most commonly applied techniques for determining the binding mode between metal complexes and DNA [47]. In this study, competitive binding experiments were carried out using ethidium bromide (EB) and Hoechst 33258 (Hoe) through fluorescence quenching measurements [48,49,50]. For the subsequent biological evaluations, complex **Pd2** was selected for detailed discussion due to its slightly higher cytotoxic activity compared to **Pd1**. Although the IC_50_ values of both complexes at 72 h are very similar, the marginally greater activity of **Pd2** allowed for a clearer illustration of the observed trends.

**Figure 5:**
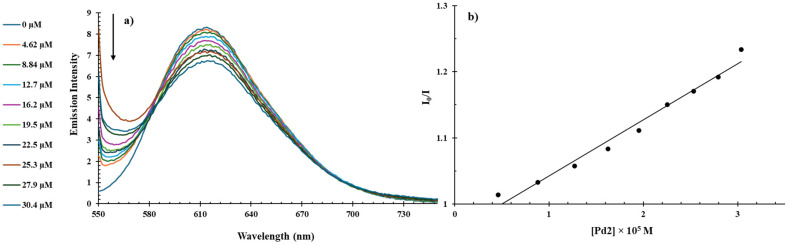
(**a**) Emission spectra of EB-DNA in the absence and presence of complex **Pd2**. pH = 7.4; λex = 550 nm; (**b**) plots of I_0_/I versus concentration of quencher [**Pd2**].

Ethidium bromide (EB) is a well-known classical intercalating agent that exhibits strong fluorescence emission upon insertion between DNA base pairs [51]. The fluorescence titration of the EB-DNA system with progressively increasing concentrations of complex **Pd2** is presented in Figure 5. The observed reduction in emission intensity at 614 nm indicates that complex **Pd2** is capable of displacing EB from the EB-DNA adduct, suggesting that it binds to DNA via an intercalative mechanism [52,53].

The Stern–Volmer quenching constants (Ksv) are calculated from the slopes of the plots of I_0_/I vs. [4] using Equation (2) [54,55]:

I_0_/I = 1 + Ksv[Q] (2)

and presented in Table 4. Quenching rate constants, kq, are calculated through the correlation (3), observing that the average fluorescence lifetime of the DNA without a quencher (τ_0_) is 10^−8^ s [56,57].

Ksv = kq × τ_0_ (3)

The obtained values for Ksv suggest that **Pd2** can interact with the DNA molecule in an intercalative mode [58].

To further elucidate the potential binding mechanism of complex **Pd2** with CT-DNA, additional competitive experiments were conducted using Hoechst 33258 (Hoe). Hoe is a synthetic derivative of N-methylpiperazine known for its ability to bind within the minor groove of DNA [59]. Minor groove binding represents a type of non-covalent interaction that is typical for many small bioactive molecules, including various therapeutic agents [60].

**Figure 6:**
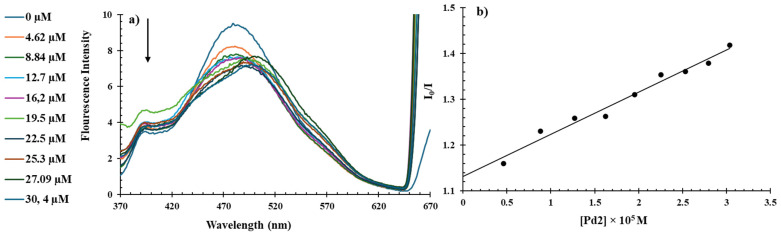
(**a**) Emission spectra of Hoe–DNA in the absence and presence of complex **Pd2**. pH = 7.4; λex = 346 nm; (**b**) plots of I_0_/I versus concentration of quencher [**Pd2**].

The fluorescence titration curve of the Hoe–DNA system upon gradual addition of complex **Pd2** is shown in Figure 6.

As illustrated in Figure 6, the observed reduction in fluorescence intensity indicates that the palladium complex **Pd2** is able to displace Hoe from the Hoe–DNA adduct. The Stern–Volmer quenching constants (Ksv) were calculated (Table 4), and the obtained values support this assumption.

Comparison of the Stern–Volmer quenching constants obtained from the EB and Hoe competitive experiments (Table 4) provides further insight into the binding behavior of complex **Pd2** toward double-stranded DNA. The results indicate that the complex is capable of interacting with DNA through both intercalative and minor groove binding modes.

Nevertheless, a bit higher Ksv value determined for the displacement of Hoe relative to that obtained for EB suggests a stronger affinity of complex **Pd2** for the minor groove region of DNA. This difference in quenching efficiency implies that, although intercalation does occur, minor groove binding represents the most common interaction pathway under the investigated experimental conditions.

#### 2.5.2. HSA Binding Studies

Serum albumin is one of the most pharmacologically significant proteins present in the bloodstream, as it plays a key role in the transport and distribution of numerous therapeutic agents to their specific target sites. Therefore, investigating the interactions between various compounds and serum albumin is essential for gaining deeper insight into the pharmacokinetic behavior and mechanism of action of potential drugs.

To investigate the binding properties of the **Pd2** complex toward serum albumin and to verify the formation of a protein complex adduct with HSA, fluorescence quenching measurements were performed.

**Figure 7:**
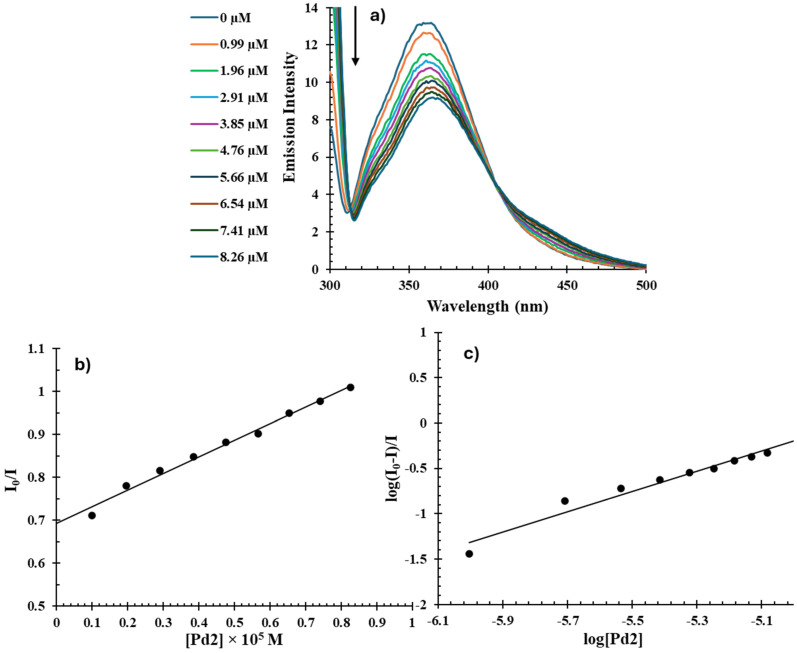
(**a**) Emission spectra of HSA in the absence and presence of **Pd2**. pH = 7.4; λex = 295 nm; (**b**) plots of I_0_/I versus [**Pd2**] in mol/dm^3^; (**c**) plots of log(I_0_ − I/I) versus log [**Pd2**].

When excited at 290 nm in buffer solution, human serum albumin (HSA) exhibits a characteristic emission maximum at 359 nm, which predominantly arises from its tryptophan residues [61]. Upon gradual addition of increasing concentrations of the **Pd2** complex to the HSA solution, a systematic reduction in fluorescence intensity was observed, as illustrated in Figure 7. This decrease in emission supports the occurrence of an interaction between the complex and the protein.

The fluorescence quenching constant (Ksv) and the bimolecular quenching rate constant (kq) were determined using the Stern–Volmer equation (Equation (2)). The calculated parameters are presented in Table 5. The obtained kq value from Equation (3) (3.9 × 10^12^ M^−1^ s^−1^) is approximately two orders of magnitude higher than the maximum value expected for a purely dynamic quenching process (2.0 × 10^10^ M^−1^ s^−1^). Such a significant difference indicates that the quenching of HSA fluorescence by the **Pd2** complex proceeds predominantly through a static mechanism, involving the formation of a ground-state complex between the protein and the quencher [62].

In cases where static quenching occurs, the dependence of fluorescence intensity on quencher concentration can be further analyzed using the Scatchard equation (Equation (4)) [63,64].

log [(I_0_ − I)/I] = log Kb + *n* · log [Q] (4)

where Kb represents the binding constant. These parameters were determined from the slope and intercept of the linear plot of log[(I_0_ − I)/I] versus log[Q], providing additional insight into the strength of the interaction between the **Pd2** complex and HSA. As the optimal values of binding constants Kb are between 10^4^ and 10^6^ M^−1^, it can be concluded that the compound **Pd2** has significant binding ability to the HSA molecule [65].

#### 2.5.3. Site-Selective Binding of the Pd2 Complex to HSA

HSA is a globular protein composed of three homologous domains (I–III), each divided into subdomains A and B [66]. The main ligand-binding regions of albumin are located in subdomains IIA (Sudlow’s site I) and IIIA (Sudlow’s site II) [67]. To determine whether the **Pd2** complex preferentially binds to site I and/or site II, competitive displacement experiments were performed using specific site markers. Eosin Y and Ibuprofen were employed as selective probes for Sudlow’s site I and site II, respectively [68,69]. The binding location of the Pd complex was evaluated by monitoring changes in the fluorescence emission of the HSA–complex system in the absence and presence of each marker individually.

**Figure 8:**
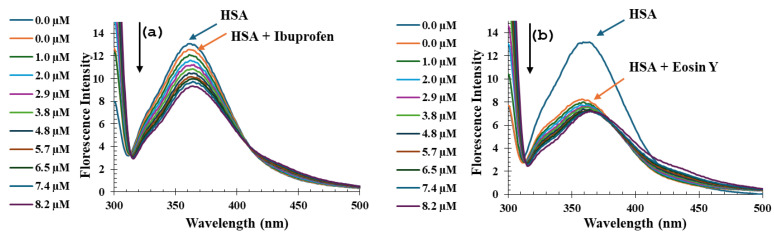
Fluorescence spectra of HSA in the absence and presence of the site marker (**a**) Ibuprofen, and (**b**) eosin Y in the presence of the various concentrations of the **Pd2** complex.

As illustrated in Figure 8a, the characteristic tryptophan emission band at 361 nm decreases upon the addition of Ibuprofen, confirming that Ibuprofen effectively occupies its binding site on HSA. When the **Pd2** complex was subsequently introduced into the Ibuprofen–HSA system, competition between the complex and Ibuprofen would be expected only if both species target the same binding region. An analogous experimental approach was applied using eosin Y (Figure 8b).

The fluorescence quenching data obtained from these competitive experiments were analyzed using the double-logarithmic equation (Equation (4)). As summarized in Table 6, the binding constant of the **Pd2** complex is significantly reduced in the presence of eosin Y, indicating competitive interaction at subdomain IIA (site I). In contrast, the binding constant remains essentially unchanged in the presence of Ibuprofen, suggesting that the complex does not compete for site II [70].

These findings demonstrate that the **Pd2** complex preferentially binds to Sudlow’s site I of HSA, most likely driven by hydrophobic interactions within subdomain IIA. Nevertheless, additional stabilizing forces, such as hydrogen bonding, electrostatic attractions, van der Waals forces, and steric effects, may also contribute to the overall binding process [71].

#### 2.5.4. Stability of the Complex **Pd2** Under Physiological Conditions

The stability of the Pd(II) complex **Pd2** under physiological conditions was evaluated spectrophotometrically in DMSO in the presence of PBS to ensure its suitability for biological studies. The UV–Vis spectra recorded over time (Figure S13, 0 h, 24 h, 48 h) showed no significant changes in the characteristic absorption bands, indicating that the complex remains stable in the tested medium. These results suggest that no substantial decomposition or structural alteration occurs under the conditions used for biological assays, confirming that the observed cytotoxic activity can be attributed to the intact complexes.

### 2.6. Molecular Basis of Dual Targeting of Catalase and Estrogen Receptor α by Pd(II) Complexes

The molecular docking analysis was performed to provide structural insight into the experimentally observed biological activity of the investigated Pd(II) complexes, which demonstrated pronounced antiproliferative effects in MCF-7 breast cancer cells and the ability to inhibit catalase activity. Given the estrogen-dependent nature of MCF-7 cells, ERα represents a biologically relevant target for evaluating potential receptor–ligand interactions associated with modulation of proliferative signaling. In parallel, CAT was included as a docking target due to its experimentally confirmed inhibition, enabling structural interpretation of enzyme–complex association at the molecular level. Molecular docking simulations were therefore carried out to evaluate the binding affinity, preferred binding orientations, and stabilizing intermolecular interactions of the Pd(II) complexes within these biologically relevant protein environments.

A comparative analysis of the docking-derived energetic parameters demonstrates that both Pd(II) complexes are capable of establishing energetically favorable interactions with the investigated protein targets. The calculated binding free energies (ΔG*_bind_*), inhibition constant (K_i_), and corresponding energetic contributions are summarized in Table 7. Both **Pd1** and **Pd2** exhibited predicted affinities within the micromolar range, indicating structurally stable association with both protein environments. Notably, **Pd2** demonstrated the most favorable overall binding profile, reaching a ΔG*_bind_* of −8.13 kcal mol^−1^ (K_i_ = 1.10 × 10^0^ µM) for CAT, while maintaining a similarly strong interaction with ERα = −7.94 kcal mol^−1^; K_i_ = 1.50 × 10^0^ µM). **Pd1** displayed a comparable trend, with ΔG*_bind_* of −8.02 kcal mol^−1^ (K_i_ = 1.33 × 10^0^ µM) for CAT and −7.58 kcal mol^−1^ (K_i_ = 2.79 × 10^0^ µM) for ERα. Although the co-crystallized ERα antagonist ligand 4-D ((2S,3R)-2-(4-(2-(piperidin-1-yl)ethoxy)phenyl)-2,3-dihydro-3-(4-hydroxyphenyl)benzo[b][1,4]oxathiin-6-ol) exhibited stronger binding within the receptor ligand-binding domain, the Pd(II) complexes demonstrated a more balanced affinity toward both investigated protein targets. In contrast, the reference CAT inhibitor 3-amino-1,2,4-triazole (3AT) displayed significantly weaker predicted binding, corresponding to substantially lower stabilization within the enzyme cavity under identical docking conditions. The comparatively favorable binding energies observed for both Pd complexes toward CAT provide structural support for their experimentally observed inhibitory activity and indicate efficient accommodation of the coordination frameworks within the catalytically relevant region of the enzyme (the structures of 3AT and 4-D are given in Figure S14).

Inspection of the energetic contributions reported in Table 7 indicates that stabilization of the Pd(II) complexes within both protein targets is primarily governed by van der Waals interactions, hydrogen bonding, and desolvation effects, while electrostatic contributions are less pronounced. This energetic profile reflects favorable steric complementarity between the coordination frameworks and the protein-binding cavities, enabling stable accommodation of the complexes within structurally compatible regions. The square-planar geometry of the Pd(II) core facilitates efficient packing within hydrophobic environments, contributing to overall complex stabilization and supporting the predicted binding affinities toward both ERα and CAT.

**Figure 9:**
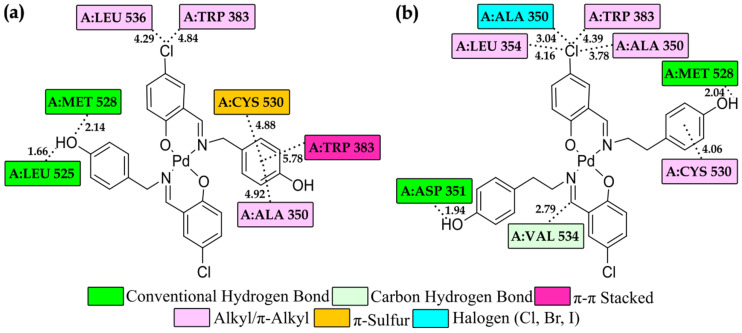
Predicted binding modes of **Pd1** (**a**) and **Pd2** (**b**) within the ligand-binding domain of human estrogen receptor alpha (ERα). Key intermolecular interactions are depicted, and selected contact distances are indicated in Å (angstrom). Interaction types are defined according to the legend.

The structural basis of the predicted interaction with ERα is illustrated in Figure 9. The representative docking conformations of **Pd1** and **Pd2** within the ERα ligand-binding domain demonstrate stable accommodation of the coordination frameworks within the receptor cavity, indicating structural compatibility with the receptor environment and supporting the favorable energetic profile observed in the docking analysis.

Detailed examination of the docking conformations reveals that both Pd(II) complexes establish multiple stabilizing interactions within the ligand-binding domain of ERα. Conventional hydrogen bonds, which play an essential role in ligand stabilization and binding specificity [72,73], are formed between the hydroxyl groups of the coordination frameworks and polar amino acid residues within the receptor cavity, including A:ASP 351, A:LEU 525, and A:MET 528. Additionally, **Pd2** forms a carbon–hydrogen bond with A:VAL 534 (Figure 9b), further contributing to stabilization and favorable orientation of the complex within the binding site.

Hydrophobic interactions also play an important role in complex stabilization. Both **Pd1** and **Pd2** establish alkyl and π–alkyl contacts with hydrophobic residues such as A:ALA 350, A:LEU 354, and A:TRP 383, supporting efficient accommodation within nonpolar regions of the receptor cavity. In addition, **Pd2** forms a halogen bond between its coordinated **Cl** atom and residue A:ALA 350, representing a directional noncovalent interaction arising from the electropositive σ-hole region of the chlorine atom and an electron-rich site of the protein backbone, further contributing to stabilization of the complex within the receptor binding site [74]. Furthermore, **Pd1** forms a π–π stacking interaction with A:TRP 383 through its aromatic moiety (Figure 9a), arising from overlap between π-electron systems and contributing to stabilization through dispersion-driven interactions [75,76]. Additional stabilization of **Pd1** is provided by a π–sulfur interaction with A:CYS 530, involving favorable contacts between the sulfur atom of the amino acid residue and the aromatic system of the coordination framework [77].

Collectively, the observed hydrogen bonding, hydrophobic contacts, and aromatic interactions provide a structural basis for stable association of the Pd(II) complexes with the ERα ligand-binding domain, supporting the predicted binding affinities and confirming structural compatibility with the receptor environment.

**Figure 10:**
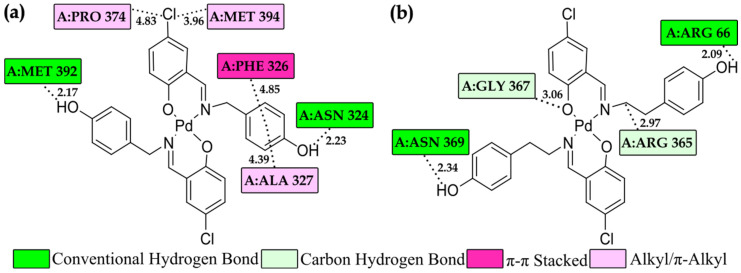
Predicted binding modes of **Pd1** (**a**) and **Pd2** (**b**) within the binding cavity of human catalase (CAT). Key intermolecular interactions are depicted, and selected contact distances are indicated in Å (angstrom). Interaction types are defined according to the legend.

The interaction of the Pd(II) complexes with CAT is illustrated in Figure 10. Both **Pd1** and **Pd2** adopt energetically favorable conformations within the enzyme binding cavity, suggesting efficient accommodation of the coordination frameworks within the protein environment. The predicted binding orientations support the favorable binding affinities obtained from docking analysis and indicate the ability of the Pd(II) complexes to establish structurally stable associations within the CAT active-site region.

Analysis of the docking conformations within CAT indicates that both Pd(II) complexes occupy well-defined regions of the enzyme binding cavity, adopting orientations that enable effective integration of the coordination frameworks within the surrounding protein environment. The square-planar geometry of the Pd(II) center allows the complexes to align favorably with the structural contours of the binding pocket, promoting stable association through multiple complementary interactions.

A significant contribution to stabilization arises from interactions with hydrophobic regions lining the enzyme cavity. The **Pd1** complex forms alkyl and π–alkyl contacts with residues such as A:PRO 374 and A:MET 394, while additional interactions involving its aromatic moiety are observed with A:ALA 327. In particular, the π–π stacking interaction with A:PHE 326 reflects favorable overlap between aromatic systems, contributing to stabilization through dispersion-driven effects and reinforcing the structural integrity of the enzyme–complex association.

Polar interactions further enhance stabilization of the complexes within the CAT environment. Hydrogen bonds are established between the hydroxyl groups of **Pd1** and **Pd2** and polar residues, including A:ARG 66, A:ASN 324, A:ASN 369, and A:MET 392, contributing to positional stabilization within the enzyme cavity. Additionally, **Pd2** forms carbon–hydrogen bonds with A:ARG 365 and A:GLY 367, supporting favorable spatial orientation and further reinforcing the stability of the bound conformation.

These combined interactions are consistent with stable accommodation of the Pd(II) complexes within the CAT binding cavity and provide a structural basis for their experimentally observed inhibitory activity, consistent with the favorable binding affinities predicted by molecular docking.

#### Molecular Basis of Pd(II) Complex Binding to Canonical and Intercalated B-Form DNA

Given the established ability of metal-based coordination compounds to interact with nucleic acids, molecular docking analysis was performed to examine the potential association of the investigated Pd(II) complexes with structurally distinct B-form DNA models. The intercalated 1Z3F and the canonical B-DNA duplex were selected to evaluate different modes of DNA association, including groove binding and intercalation-associated interactions. The calculated energetic parameters describing the predicted DNA–complex interactions are summarized in Table 8.

The docking results reveal favorable binding of both Pd(II) complexes to B-form DNA, with a pronounced preference for intercalated 1Z3F. In particular, **Pd2** exhibited the strongest predicted affinity, reaching a ΔG*_bind_* of −9.71 kcal mol^−1^, corresponding to a K_i_ of 0.08 µM. **Pd1** also demonstrated substantial stabilization within the same DNA environment, with a ΔG*_bind_* of −8.63 kcal mol^−1^. These results indicate efficient accommodation of the coordination frameworks within DNA structures that permit intercalation-associated binding modes.

In contrast, interactions with the canonical B-DNA duplex were characterized by moderately lower binding affinities, with **Pd1** exhibiting a ΔG*_bind_* of −7.51 kcal mol^−1^ and **Pd2** −6.45 kcal mol^−1^. This energetic distinction suggests that the structural environment of 1Z3F provides more favorable conditions for stabilization of the Pd(II) coordination frameworks.

According to the energetic contributions presented in Table 8, stabilization of the Pd(II) complexes within both DNA models is predominantly controlled by dispersion interactions, hydrogen bonding, and desolvation effects, with comparatively minor electrostatic contributions. This energetic profile reflects favorable structural complementarity between the coordination frameworks and the DNA environment, enabling stable association through interactions with nucleoside residues and surrounding regions of the double helix. These findings demonstrate the ability of the investigated Pd(II) complexes to establish structurally stable interactions with DNA.

**Figure 11:**
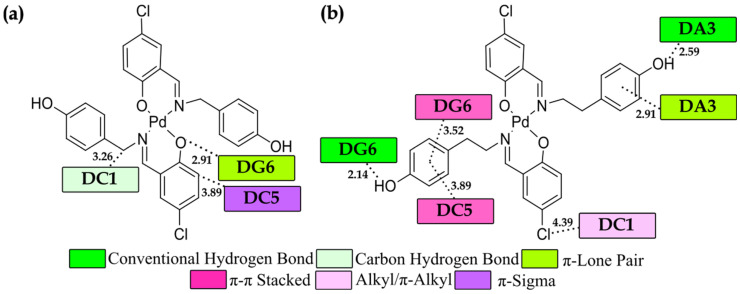
Predicted binding modes of **Pd1** (**a**) and **Pd2** (**b**) within the intercalated B-form DNA duplex (1Z3F). Key intermolecular interactions are depicted, and interacting nucleosides are labeled as DC (deoxycytidine), DA (deoxyadenosine), and DG (deoxyguanosine). Selected contact distances are indicated in Å (angstrom), and interaction types are defined according to the legend.

The structural determinants underlying the predicted interaction with the intercalated 1Z3F are illustrated in Figure 11. The representative docking conformations suggest stable positioning of the coordination frameworks within regions compatible with intercalation-associated binding. The observed orientations enable effective spatial complementarity with surrounding nucleoside residues, facilitating stabilizing intermolecular contacts and supporting the favorable energetic profile obtained from docking analysis.

The 1Z3F provides a structurally favorable environment for evaluating the ability of the Pd(II) complexes to establish stabilizing interactions within regions compatible with intercalation-associated binding. Within this framework, **Pd2** forms conventional hydrogen bonds with nucleoside residues DA3 and DG6 through its hydroxyl groups, contributing to positional stabilization of the coordination framework within the DNA structure. In parallel, **Pd1** establishes a carbon–hydrogen bond with DC1, further supporting structural stabilization and favorable alignment relative to adjacent nucleosides.

In addition to hydrogen bonding, both complexes engage in stabilizing noncovalent interactions with surrounding nucleoside residues. The coordination environment of **Pd2** enables alkyl and π–alkyl contacts with DC1, facilitating efficient accommodation within the DNA environment. Furthermore, the aromatic moiety of **Pd2** participates in π–π stacking interactions with DC5 and DG6, arising from overlap between aromatic π-electron systems and contributing to stabilization through dispersion-driven effects. Additional stabilizing contributions arise from interactions involving aromatic electron density and neighboring structural elements. π–sigma interactions are observed between **Pd1** and DC5, reflecting favorable overlap between aromatic π-systems and adjacent σ-bonds [77]. Moreover, π–lone pair interactions [77,78] are identified between DG6 and the oxygen atom of **Pd1**, as well as between DA3 and the aromatic moiety of **Pd2**, further enhancing structural compatibility between the coordination frameworks and the DNA structure.

Collectively, the combination of hydrogen bonding and multiple aromatic and hydrophobic interactions enables stable accommodation of the Pd(II) complexes within the 1Z3F environment, supporting the favorable binding affinities predicted by docking analysis.

**Figure 12:**
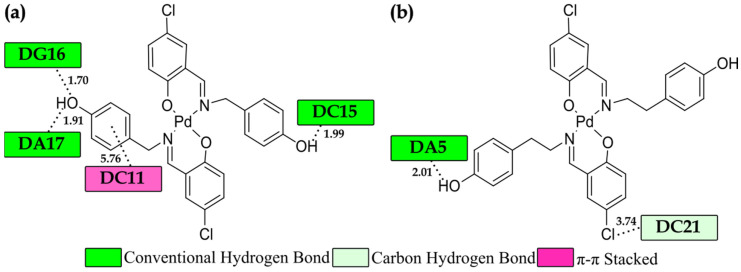
Predicted binding modes of **Pd1** (**a**) and **Pd2** (**b**) within the canonical B-form DNA duplex (B-DNA). Key intermolecular interactions are depicted, and interacting nucleosides are labeled as DC (deoxycytidine), DA (deoxyadenosine), and DG (deoxyguanosine). Selected contact distances are indicated in Å (angstrom), and interaction types are defined according to the legend.

Docking conformations obtained for the canonical B-DNA duplex indicate that the Pd(II) complexes preferentially associate with groove-accessible regions of the double helix, rather than inserting between adjacent nucleosides. As illustrated in Figure 12, both complexes adopt energetically favorable orientations along the DNA structure, enabling stabilizing interactions with exposed nucleoside residues and surrounding elements of the DNA backbone. The coordination frameworks appear to be accommodated in structurally accessible regions of the helix, supporting stable ligand–DNA association and reflecting the binding affinities predicted by docking analysis.

Examination of the docking conformations within the B-DNA reveals that both Pd(II) complexes establish stabilizing interactions with nucleoside residues located along groove-accessible regions of the double helix. Aromatic and hydrophobic interactions play an important role in the stabilization of the coordination frameworks within the DNA environment. In particular, **Pd1** forms a π–π stacking interaction with nucleoside DC11 via its aromatic moiety (Figure 12a). This interaction arises from overlap between aromatic π-electron systems and contributes to stabilization through dispersion-driven effects, supporting favorable accommodation of the coordination framework along the DNA groove.

Additional structural support for **Pd2** is achieved through the formation of a carbon–hydrogen bond with DC21, further reinforcing the favorable positioning of the coordination framework relative to adjacent nucleosides (Figure 12b). Hydrogen bonding interactions also contribute significantly to structural stabilization, with conventional hydrogen bonds observed between the hydroxyl groups of the Pd(II) complexes and nucleosides DA5, DC15, DG16, and DA17. These interactions help anchor the coordination frameworks within accessible regions of the DNA structure and support stable ligand–DNA association.

The combined contribution of aromatic interactions, hydrophobic contacts, and hydrogen bonding enables stable accommodation of the Pd(II) complexes along the B-DNA, consistent with the predicted binding affinities obtained from docking analysis.

Collectively, the docking analysis reveals that the investigated Pd(II) complexes exhibit a pronounced ability to establish stable and energetically favorable interactions with ERα, CAT, and B-form DNA structures. The predicted binding modes suggest effective structural accommodation of the coordination frameworks within protein-binding cavities and DNA environments. The particularly favorable interactions observed with CAT and 1Z3F highlight the potential structural compatibility of the Pd(II) coordination cores with biologically relevant molecular environments. These findings provide a structural context for the experimentally observed cytotoxic activity in MCF-7 cells and CAT inhibition, supporting the ability of the complexes to interact with multiple biomolecular targets. Such a dual protein–DNA interaction profile underscores the potential of the investigated Pd(II) complexes to engage diverse molecular components associated with cancer cell function and oxidative stress regulation.

Although the synthesized palladium(II) complexes demonstrated promising in vitro anticancer activity and dual-targeting potential toward catalase and DNA, this study is limited to cellular and computational models. Future investigations should focus on in vivo evaluation, deeper mechanistic studies, and structural optimization to enhance selectivity, potency, and therapeutic efficacy. The MCF7 cell line was employed as an initial and widely used in vitro model for preliminary assessment of anticancer activity; nevertheless, broader validation across multiple cancer cell lines will be necessary in future studies to confirm the generality of the observed effects. In addition, as antitumor activity was assessed in a single cell line and ROS levels were not directly evaluated, a comprehensive correlation analysis between these parameters was beyond the scope of the present study and remains to be addressed in future investigations.

## 3. Materials and Methods

### 3.1. General Procedures and Materials

All chemicals were commercially available and were used as received, without further purification. NMR spectra were recorded in DMSO-*d*_6_ as the solvent using a Varian Bruker Avance III 300 MHz spectrometer (Billerica, MA, USA). Elemental analyses (C, H, O, N) were performed with an Elementar (Langenselbold, Germany) Vario MICRO elemental analyzer. IR spectra were collected using a PerkinElmer (Waltham, MA, USA) FT-IR spectrometer equipped with a DTGS detector. Mass spectra were analyzed using a high-resolution 6546 LC/Q-TOF mass spectrometer (Agilent, Santa Clara, CA, USA) coupled to a 1290 Infinity II HPLC liquid chromatograph (Agilent, Santa Clara, CA, USA). The samples were dissolved in a mixture of 100% methanol and 100% acetonitrile (1:1) and then analyzed. Sample spectra were acquired under positive ionization in high-resolution positive mode. Data were collected every second over a mass range of 100–1000 Da. Molar conductance of the Pd(II) complexes was measured on freshly prepared 10^–3^ M solutions in DMSO at room temperature using Crison (Alella, Spain) EC-Meter basic 30+conductivity cell.

Dulbecco’s Modified Eagle Medium for cell culturing (DMEM), MTT (4,5-dimethylthiazol-2-yl)-2,5-diphenyltetrazolium bromide) and dimethyl sulfoxide (DMSO) were obtained from GIBCO, Invitrogen, Norristown, PA, USA. Ethidium bromide (EA) was obtained from SERVA, Germany, and acridine orange (AO) from Acros Organics, Fair Lawn, NJ, USA.

HAS/DNA solutions were prepared in 0.01 M phosphate-buffered saline buffer at pH = 7.4. The fluorescence spectroscopy on an RF-1501 PC spectrofluorometer (Shimadzu, Tokyo, Japan) was used for analysis of the results of the interaction between HAS/DNA and the tested compound.

#### 3.1.1. General Procedure for the Synthesis of Schiff Base Ligands

*p*-Hydroxybenzylamine/or tyramine (1 mmol) was dissolved in 15 mL of absolute ethanol, after which an equimolar amount (1 mmol) of the 5-chlorosalicylic aldehyde was added. The reaction mixture was stirred at room temperature overnight. Upon completion of the reaction, the resulting solid products were collected by filtration, washed with cold ethanol, and dried under vacuum. The corresponding spectra (Supplementary Material, Figures S1–S4) for compound **1** are provided in the Supplementary Material.

#### 3.1.2. General Procedure for the Synthesis of **Pd1** and **Pd2**

One millimole of the appropriate ligand was dissolved in 10 mL of acetonitrile, after which 0.5 mmol of PdCl_2_ was added. The reaction mixture was stirred at room temperature for 30 min, leading to the formation of orange to red–orange precipitates. The resulting solids were isolated by filtration, washed with cold acetonitrile, and dried under vacuum.

(*E*)-4-chloro-2-(((4-hydroxybenzyl)imino)methyl)phenol **1**. Yellow powder, yield 86%, ^1^H NMR (300 MHz, DMSO-*d*_6_): δ 13.57 (s, 1H, -O-H), 9.42 (s, 1H, -O-H), 8.63 (s, 1H, -N=C-H), 7.56 (d, *J* = 2.7 Hz, 1H, Ar-H), 7.34 (dd, *J* = 8.8, 2.7 Hz, 1H, Ar-H), 7.24–7.05 (m, 2H, Ar-H), 6.89 (d, *J* = 8.8 Hz, 1H, Ar-H), 6.82–6.57 (m, 2H, Ar-H), 4.68 (s, 2H, -CH_2_-). ^13^C NMR (75 MHz, DMSO-*d_6_*): δ 164.70 (-C=N-), 159.82, 156.71, 132.07, 130.62, 129.24, 128.35, 121.78, 119.71, 118.67, 115.38, 61.41. IR (cm^−1^): 1644 (ν(C=N). HRMS (ESI^+^): *m*/*z* calculated for C_14_H_12_ClNO_2_ [M + H] ^+^ 262.0629; found 262.0630. Anal. Found: C, 63.74; H, 4.71; N, 5.29. Calc. for C_14_H_12_ClNO_2_: C, 64.25; H, 4.62; N, 5.35; O, 12.23.

(*E*)-4-Chloro-2-((4-hydroxyphenethylimino)methyl) phenol **2**. Orange crystals, yield 93%. ^1^H NMR (300 MHz, DMSO-*d_6_*): δ 13.61 (s, 1H, -O-H), 9.21 (s, 1H, -O-H), 8.43 (s, 1H,-N=C-H), 7.47 (d, *J* = 2.7 Hz, 1H, Ar-H), 7.32 (dd, *J* = 8.8, 2.7 Hz, 1H, Ar-H), 7.02 (d, *J* = 8.5 Hz, 2H, Ar-H), 6.87 (d, *J* = 8.8 Hz, 1H, Ar-H), 6.67 (d, *J* = 8.5 Hz, 2H, Ar-H), 3.77 (t, *J* = 6.8 Hz, 2H, -CH_2_-), 2.82 (t, *J* = 7.0 Hz, 2H, -CH_2_-). ^13^C NMR (75 MHz, DMSO-*d*_6_): δ 164.97, 160.25 (-C=N-), 155.87, 132.17, 130.68, 129.91, 129.41, 121.74, 119.68, 118.92, 115.33, 60.02, 35.89.

Complex **Pd1**. Yellow powder, yield 72%. ^1^H NMR (300 MHz, DMSO-*d*_6_): δ 9.62 (s, 1H, -OH), 8.01 (s, 1H,-N=C-H), 7.64–7.47 (m, 2H, Ar-H), 7.25 (d, *J* = 8.0 Hz, 2H, Ar-H), 6.77 (t, *J* = 9.3 Hz, 2H, Ar-H), 3.89 (d, *J* = 5.3 Hz, 2H, -CH_2_-). ^13^C NMR (75 MHz, DMSO-*d*_6_): δ 189.88, 159.73 (-C=N-), 157.90, 135.97, 130.73, 129.53, 127.52, 124.27, 123.52, 119.74, 118.35, 115.52, 42.18. HRMS (ESI^+^): *m*/*z* calculated for C_28_H_22_Cl_2_N_2_O_4_Pd [M + H]^+^ 629.0059 (100%); found 629.0054. Anal. Calcd for C_28_H_22_Cl_2_N_2_O_4_Pd: C, 53.57; H, 3.53; N, 4.46; O, 10.19. Found: C, 53.68; H, 3.41; N, 4.59.

Complex **Pd2**. Yellow powder, yield 79%. ^1^H NMR (300 MHz, DMSO-*d*_6_): δ 9.23 (s, 1H, -OH), 7.89 (s, 1H, -N=C-H), 7.36 (d, *J* = 2.8 Hz, 1H, Ar-H), 7.31–7.22 (m, 1H, Ar-H), 7.07 (d, *J* = 8.5 Hz, 2H, Ar-H), 6.79 (d, *J* = 9.0 Hz, 1H, Ar-H), 6.68 (d, *J* = 8.5 Hz, 2H, Ar-H), 3.84 (t, *J* = 7.3 Hz, 2H, -CH_2_-), 2.90 (t, *J* = 7.4 Hz, 2H, -CH_2_-). ^13^C NMR (75 MHz, DMSO-*d*_6_): δ 162.53 (-C=N-), 162.12, 155.78, 134.24, 132.72, 129.81, 128.61, 121.50, 121.11, 117.86, 115.23, 58.67. HRMS (ESI^+^): *m*/*z* calculated for C_30_H_26_Cl_2_N_2_O_4_Pd [M + H] 657.0373 (100%), found 657.0373. Anal. Calcd for C_30_H_26_Cl_2_N_2_O_4_Pd: C, 54.94; H, 4.00; N, 4.27; O, 9.76. Found: C, 54.88; H, 4.12; N, 4.34.

### 3.2. Cell Culturing

The breast cancer cell line (MCF-7) was obtained from the American Tissue Culture Collection (Manassas, VA, USA), maintained in laboratory conditions, and prepared for experiments according to standard protocols [79].

#### 3.2.1. Cytotoxic Activity—MTT Assay

Determination of Pd complexes cytotoxicity on MCF-7 cancer cells was conducted using MTT assay [80], based on the reduction in MTT to formazan crystals by metabolically active (viable) cells. Dissolved crystals in DMSO were solubilized and quantified spectrophotometrically at 550 nm. For this assay, cells were seeded in a 96-well plate (10^4^ cells/well), after incubation for 24 h, followed by treatments with Pd complexes in a concentration range of 1–200 µM, and medium only for the control cells. The result was expressed initially across cell viability in all applied concentrations after 24 and 72 h, where the control absorbances were considered as 100% cell viability, and then the cytotoxicity was assessed based on calculated IC50 values in the CalcuSyn program.

#### 3.2.2. Cell Morphology Analysis

Cell morphology of MCF-7 control and cells treated with 20 µM concentration of Pd complexes on a fluorescent microscope was observed after double staining with acridine orange/ethidium bromide (AO/EB) [81]. The morphology of cells and different colored fluorescent signals were considered in the analysis. Based on this observation and micrographs of samples, the predominant type of cell death was determined. For this assay, cells were seeded in a 96-well plate (10^4^ cells/well), incubated overnight, and then treated for 24 h. The cells were observed under the fluorescent microscope (Ti-Eclipse, Nikon, Tokyo, Japan) at 400× magnification immediately after the addition of the dyes.

#### 3.2.3. Statistical Analysis

All data are expressed as mean ± standard error (SE) and were obtained in three individual experiments. Statistical significance was determined by Student’s *t*-test for comparisons of control values vs. values in treatments. A *p*-value < 0.05 was considered significant. The magnitude of the correlation between variables was calculated using software (SPSS for Windows, ver. 17, 2008, Chicago, IL, USA).

### 3.3. Catalase Inhibition

The inhibition of catalase was examined using a spectrophotometric method, in which the obtained absorbance values were converted into corresponding reaction rates based on kinetic principles.

To determine the effect of palladium complexes on catalase activity, the Michaelis–Menten equation was used. Measurements were carried out at 240 nm and at a temperature of 37 °C. The reaction mixture (total volume 130 μL) consisted of phosphate buffer pH 7.00 (100 mM), 10 μL of enzyme solution, various concentrations of hydrogen peroxide ranging from 0.0015 M to 0.0076 M, and three different concentrations of Pd complex: 0.07 mg/mL, 0.11 mg/mL, and 0.25 mg/mL.

### 3.4. Albumin-Binding Studies

The protein-binding studies were made by using a fixed HSA concentration (2 µM) and varying the concentration of the analyzed compound (0–9.1 µM). The site competitive replacement experiments were performed in the presence of two site markers (eosin Y and Ibuprofen) using the fluorescence titration methods to identify the binding location of the **Pd2** complex on HSA. Equimolar concentrations (2 µM) of HSA and eosin Y/Ibuprofen were used, and the **Pd2** complex was then added gradually. Spectra were recorded in the range between 300 and 500 nm, with an excitation wavelength of 295 nm.

#### DNA-Binding Studies

Competitive binding experiments involving EB and Hoe were carried out for complex **Pd2** using fluorescence emission spectroscopy. The DNA–EB and DNA–Hoe systems were prepared by combining 0.1 mM EB or Hoe with 2 mM CT-DNA in phosphate-buffered saline (PBS) at pH 7.4.

Fluorescence measurements were performed immediately after the stepwise addition of complex **Pd2** (0–30 µM) to the preformed DNA–EB or DNA–Hoe solutions in order to evaluate the potential binding interactions of the investigated compound. The excitation wavelength was set at 527 nm for EB and 346 nm for Hoe. Emission spectra were recorded over the range of 550–750 nm for the EB system and 360–600 nm for the Hoe system.

### 3.5. Computational Investigation of Pd(II) Complexes: Density Functional Theory Optimization and Molecular Docking Analysis

While experimental studies demonstrated significant cytotoxic activity of the investigated Pd(II) complexes against MCF-7 breast cancer cells, as well as their ability to inhibit catalase activity, the molecular basis of these effects is governed by structure-dependent interactions at the atomic level. To examine such interactions from a structural perspective, molecular docking simulations were performed using the DFT-optimized geometries of the Pd(II) complexes. The objective of the computational analysis was to characterize potential binding orientations, evaluate spatial complementarity with selected macromolecular targets, and identify key intermolecular contacts relevant to ligand–target association.

The molecular geometries of the Pd(II) complexes were optimized by density functional theory (DFT) calculations using the Gaussian16 program package [82] at the B3LYP-D3BJ level of theory. The 6-311+G(d,p) basis set was applied for C, H, N, O, and Cl atoms, while the Pd center was described using the LANL2DZ basis set together with the corresponding effective core potential [83]. Vibrational frequency analysis confirmed the absence of imaginary frequencies, verifying that the optimized geometries correspond to a true minimum on the potential energy surface. These optimized structures were subsequently used as input geometries for molecular docking simulations. The crystallographic structures of human estrogen receptor alpha (ERα, PDB ID: 1SJ0) [84] and human catalase (CAT, PDB ID: 1QQW) [85] were obtained from the RCSB Protein Data Bank and used as enzymatic targets. The intercalated DNA duplex (PDB ID: 1Z3F) [86] and the canonical B-DNA dodecamer d(CGCGAATTCGCG)_2_ (PDB ID: 1BNA) [87] were retrieved from the same source and used as nucleic acid targets. Macromolecular target preparation was performed using BIOVIA Discovery Studio 4.0 [88]. Crystallographic water molecules, buffer components, and non-essential heteroatoms were removed to ensure a consistent representation of the binding environments. Polar hydrogen atoms were added, and Kollman united-atom charges were assigned according to the standard AutoDock protocol. For CAT, the heme prosthetic group was retained due to its structural role within enzyme architecture. DNA structures were prepared using the same procedure, preserving the integrity of the double-helical framework. All macromolecular targets were treated as rigid structures during docking calculations. Ligands were prepared according to the same protocol. Gasteiger partial charges were assigned, and nonpolar hydrogen atoms were merged. Conformational flexibility was allowed within the organic ligand framework, while the square-planar Pd(II) coordination geometry was preserved to maintain consistency with the DFT-optimized structures. Molecular docking simulations were performed using AutoDock Tools 1.5.7 and AutoDock 4.2.6 [89,90]. The conformational space of the ligands was explored employing the Lamarckian Genetic Algorithm (LGA) [90,91]. Grid boxes were defined as encompassing the relevant binding regions of each target structure. For the canonical B-DNA duplex, the grid center was positioned at coordinates 15.81 × 21.31 × 9.88 Å, and a grid box of dimensions 60 × 74 × 120 Å was used to cover the entire DNA structure. The genetic algorithm parameters included a population size of 150 individuals, a maximum of 2,500,000 energy evaluations, and 27,000 generations per run, with mutation and crossover rates set to 0.02 and 0.8, respectively. Multiple independent docking runs were performed to ensure reproducibility and convergence of the predicted binding conformations.

Docked conformations were ranked according to predicted binding free energies calculated using the AutoDock empirical free energy scoring function. The most favorable binding poses were further analyzed using BIOVIA Discovery Studio 4.0 [88] to identify hydrogen bonding, hydrophobic interactions, π-interactions, electrostatic contributions, and overall structural complementarity between the Pd(II) complexes and the investigated targets.

## 4. Conclusions

In this study, two novel palladium(II) complexes derived from salicylic Schiff base ligands containing chloro-substituted salicylaldehyde moieties were successfully synthesized and structurally characterized using spectroscopic techniques, mass spectrometry, and elemental analysis. The obtained results confirmed the coordination of the ligands to the Pd(II) center through the imine nitrogen and phenolic oxygen atoms, leading to the formation of stable square-planar complexes with a 1:2 metal-to-ligand stoichiometry.

Biological evaluation revealed that both complexes exhibited pronounced cytotoxic activity against the MCF-7 breast cancer cell line in a dose- and time-dependent manner, particularly **Pd2**. Morphological analysis of treated cells showed that cytotoxicity was achieved by apoptosis, as indicated by morphological changes, while necrosis was present in traces. The inhibition studies of liver catalase demonstrated that both Pd complexes act as uncompetitive inhibitors, binding preferentially to the enzyme–substrate complex and significantly affecting the catalytic activity of catalase. This inhibition may contribute to increased intracellular oxidative stress through the accumulation of hydrogen peroxide and elevated levels of reactive oxygen species, which can promote apoptotic pathways in cancer cells. Fluorescence quenching experiments further revealed that complex **Pd2** interacts with CT-DNA through a combination of intercalative and minor groove binding modes, with a slightly stronger affinity toward the minor groove region. Additionally, the complex exhibited significant binding affinity toward human serum albumin, with fluorescence quenching analysis indicating a predominantly static mechanism of interaction and formation of a stable protein–complex adduct. Competitive binding studies demonstrated that **Pd2** preferentially associates with Sudlow’s site I within subdomain IIA of HSA. Molecular docking analysis provided structural insight into the experimentally observed biological effects. The investigated Pd(II) complexes demonstrated favorable binding affinities toward catalase, estrogen receptor α, and B-form DNA models. The predicted interactions were primarily stabilized by van der Waals forces, hydrogen bonding, and hydrophobic contacts, enabling efficient accommodation of the coordination frameworks within the corresponding biomolecular environments. Notably, **Pd2** exhibited the most favorable docking profile toward catalase and intercalated DNA structures, supporting the experimentally observed biological activity.

Overall, the combined experimental and computational results demonstrate that the synthesized palladium(II) complexes possess significant antiproliferative activity and the ability to interact with multiple biologically relevant targets, including catalase, DNA, and serum albumin. These findings highlight the potential of salicylic Schiff base-derived Pd(II) complexes as promising candidates for further development of metal-based anticancer agents. Future studies should focus on detailed mechanistic investigations, evaluation in additional cancer models, and assessment of their in vivo therapeutic potential.

## Figures and Tables

**Table 1:** Characteristic NMR chemical shifts and IR absorption bands of free ligand compounds and corresponding palladium complexes.

Ligand/Complex	IR abs. Bands (cm^−1^)	NMR Shifts (ppm)	
	C=N	C=N	OH
**1**	1644	8.63	13.57
**Pd1**	1625	7.99	Disappeared
**2**	1625	8.44	13.64
**Pd2**	1621	7.89	Disappeared

**Table 2:** The IC_50_ values (µM) represent the cytotoxicity of Pd complexes on MCF-7 cells.

Treatments	24 h	72 h
**Pd1**	48.6 ± 1.6	46.4 ± 1.7
**Pd2**	63.2 ± 1.5	40.7 ± 1.0
**Cisplatin**	5.75 ± 0.02 [40]	10.70 µM ± 2.09 µM [41]

**Table 3:** Predicted lipophilicity (Log *P*_o/w_) of **Pd1** and **Pd2** complexes and **Pd1** chloro-free model.

Lipophilicity	Pd1 (Chloro-Free Model Structure)	Pd1	Pd2
Log *P*_o/w_ (iLOGP)	0.00	0.00	0.00
Log *P*_o/w_ (XLOGP3)	5.11	6.36	6.96
Log *P*_o/w_ (WLOGP)	4.64	5.95	6.34
Log *P*_o/w_ (MLOGP)	2.26	3.18	3.56
Log *P*_o/w_ (SILICOS-IT)	1.96	3.23	4.03
Consensus Log *P*_o/w_	2.79	3.74	4.18

**Table 4:** The bimolecular quenching rate constant (kq), Stern–Volmer constant (Ksv), and correlation coefficient (*R*) for complex **Pd2** as a quencher.

Complex	Test	kq [M^−1^ s^−1^]	Ksv [M^−1^]	*R*
**Pd2**	EB	8.5 × 10^11^	8.5 × 10^3^	0.9758
	Hoe	9.2 × 10^11^	9.2 × 10^3^	0.9753

**Table 5:** The bimolecular quenching rate constant (kq), Stern–Volmer constant (Ksv), the binding parameter (Kb), and the correlation coefficient (*R*) for the interaction of the tested compound with HSA.

Compound	kq [M^−1^ s^−1^]	*Ksv* [M^−1^]	*R*	Kb [M^−1^]	*R*
**Pd2**	3.9 × 10^12^	3.9 × 10^4^	0.989	2.5 × 10^5^	0.956

**Table 6:** Binding constant (Kb) and correlation coefficient (*R*) for the interaction of the **Pd2** complex with HSA in the absence and presence of the site marker probes.

System	Kb [M^−1^]	*R*
**Pd2**–HSA	2.5 × 10^5^	0.956
**Pd2**–HSA–Ibuprofen	6.8 × 10^4^	0.998
**Pd2**–HSA–Eosin Y	1.1 × 10^1^	0.984

**Table 7:** Molecular docking binding parameters of **Pd1** and **Pd2** complexes and reference inhibitors within the ligand-binding domains of human estrogen receptor alpha (ERα) and human catalase (CAT). The calculated energetic profile of the docking-derived complex–protein associations is summarized in the table, including the predicted binding free energy (Δ*G*_bind_, kcal mol^−1^) and the corresponding inhibition constant (*K*_i_, µM). Furthermore, the individual energetic contributions to the overall binding process are reported, comprising the total internal energy (Δ*G*_total_), torsional energy (Δ*G*_tor_), unbound system energy (Δ*G*_unb_), electrostatic interactions (Δ*G*_elec_), the sum of dispersion and repulsion forces (Δ*G*_vdw_), hydrogen bonding (Δ*G*_hbond_), and desolvation energy (Δ*G*_desolv_).

Complexes	ΔG*_bind_*	K_i_ (µM)	ΔG*_inter_*	ΔG*_vdw_*_+*hbond*+*desolv*_	ΔG*_elec_*	ΔG*_total_*	ΔG*_tor_*	ΔG*_unb_*
ERα								
**Pd1**	−7.58	2.79 × 10^0^	−9.22	−9.08	−0.14	−1.34	1.65	−1.34
**Pd2**	−7.94	1.50 × 10^0^	−10.14	−9.82	−0.32	−2.84	2.20	−2.84
4-D	−12.48	7.09 × 10^−4^	−14.68	−14.35	−0.32	−1.55	2.20	−1.55
CAT								
**Pd1**	−8.02	1.33 × 10^0^	−9.66	−9.66	−0.00	−1.37	1.65	−1.37
**Pd2**	−8.13	1.10 × 10^0^	−10.33	−10.29	−0.03	−1.84	2.20	−1.84
3AT	−3.95	1.27 × 10^0^	−4.25	−3.08	−1.17	0.11	0.30	0.11

**Table 8:** Molecular docking binding parameters of **Pd1** and **Pd2** complexes with the intercalated B-form DNA duplex (1Z3F) and canonical B-form DNA duplex (B-DNA). The calculated energetic profile of the docking-derived complex–DNA associations is summarized in the table, including the predicted binding free energy (Δ*G*_bind_, kcal mol^−1^) and the corresponding inhibition constant (*K*_i_, µM). Furthermore, the individual energetic contributions to the overall binding process are reported, comprising the total internal energy (Δ*G*_total_), torsional energy (Δ*G*_tor_), unbound system energy (Δ*G*_unb_), electrostatic interactions (Δ*G*_elec_), the sum of dispersion and repulsion forces (Δ*G*_vdw_), hydrogen bonding (Δ*G*_hbond_), and desolvation energy (Δ*G*_desolv_).

Complexes	ΔG*_bind_*	K_i_ (µM)	ΔG*_inter_*	ΔG*_vdw_*_+*hbond*+*desolv*_	ΔG*_elec_*	ΔG*_total_*	ΔG*_tor_*	ΔG*_unb_*
1Z3F								
**Pd1**	−8.63	0.47	−10.28	−9.97	−0.30	−0.82	1.65	−0.82
**Pd2**	−9.71	0.08	−11.91	−11.44	−0.46	−1.43	2.20	−1.43
B-DNA								
**Pd1**	−7.51	3.12	−9.16	−8.78	−0.38	−1.69	1.65	−1.69
**Pd2**	−6.45	18.69	−8.65	−8.53	−0.12	−2.54	2.20	−2.54

## Data Availability

The original contributions presented in this study are included in the article/Supplementary Material. Further inquiries can be directed to the corresponding author.

## Supplementary Materials

The following supporting information can be downloaded at https://www.mdpi.com/article/10.3390/molecules31081370/s1.
