# Supplementary material for: Palladium(II) Complexes with Chloro-Substituted Salicyl Schiff Bases: Exploring Multimodal Anticancer Mechanisms and Catalase Inhibition
Source: Molecules. 2026 Apr 21;31(8):1370. doi: 10.3390/molecules31081370 (PMC13119235; doi:10.3390/molecules31081370)
Supplement: Supplementary file 1 [file molecules-31-01370-s001.zip › molecules-4234018-supplementary.pdf]

Supplementary Information

Palladium(II) Complexes with Chloro-Substituted Salicyl Schiff Bases: Exploring Multimodal Anticancer Mechanisms and Catalase Inhibition

Jovana S. Dragojević [1], Žiko Milanović [2], Kristina Milisavljević [2], Milena Milutinović [3], Safija Herenda [4], Edhem Hasković [5], Nenad Vanis [6], Vera M. Divac [1] and Marina D. Kostić [2,*]

1 Department of Chemistry, Faculty of Science, University of Kragujevac, Radoja Domanovića 12, 34000 Kragujevac, Serbia; jovana.marjanovic@pmf.kg.ac.rs (J.S.D.); vera.divac@pmf.kg.ac.rs (V.M.D.)

2 Department of Science, Institute for Information Technologies, University of Kragujevac, Liceja Kneževine Srbije 1A, 34000 Kragujevac, Serbia; ziko.milanovic@uni.kg.ac.rs (Ž.M.); kristina.milisavljevic@uni.kg.ac.rs (K.M.)

3 Department of Biology and Ecology, Faculty of Science, University of Kragujevac, Radoja Domanovića 12, 34000 Kragujevac, Serbia; milena.milutinovic@pmf.kg.ac.rs

4 Department of Chemistry, Faculty of Science, University of Sarajevo, 71000 Sarajevo, Bosnia and Herzegovina; safija@pmf.unsa.ba

5 Department of Biology, Faculty of Science, University of Sarajevo, 71000 Sarajevo, Bosnia and Herzegovina; e.haskovic@pmf.unsa.ba

6 ASA Hospital, Džemala Bijedica 127, 71000 Sarajevo, Bosnia and Herzegovina; nenad.vanis@ssst.edu.ba

* Correspondence: marinak@uni.kg.ac.rs

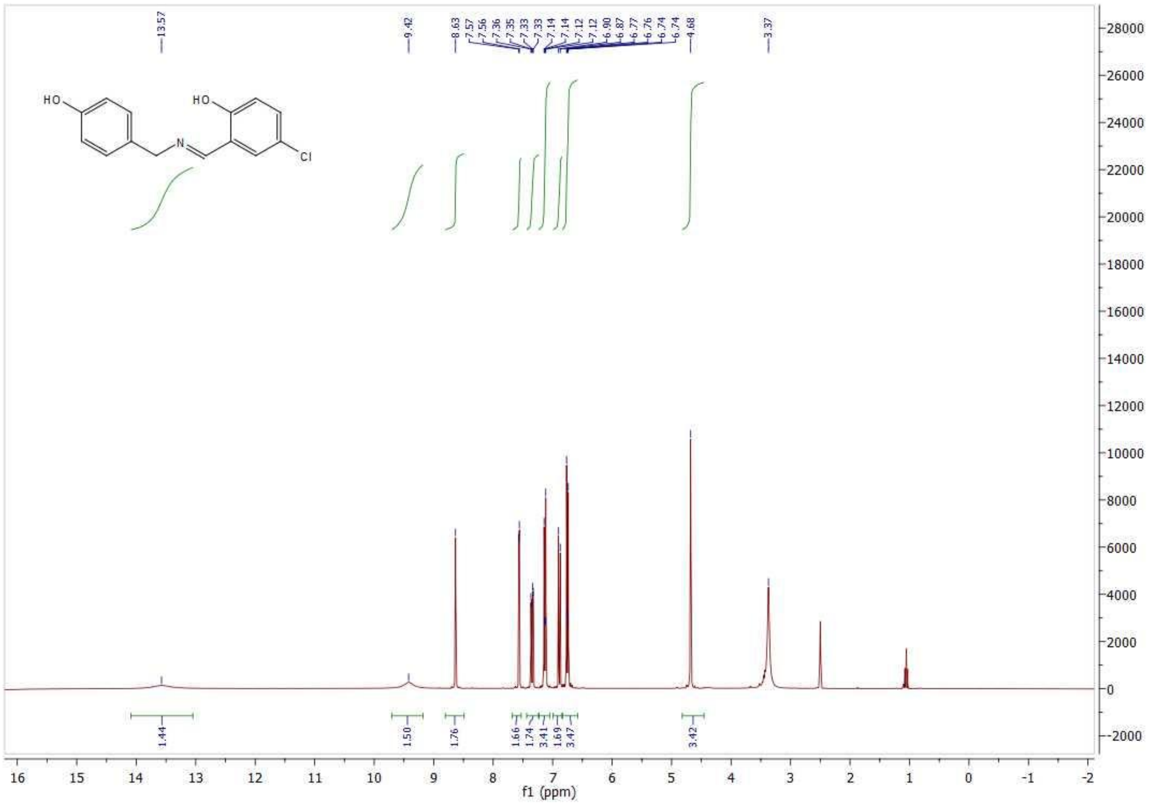

Figure S1. ^{1}H NMR spectra of compound **1**

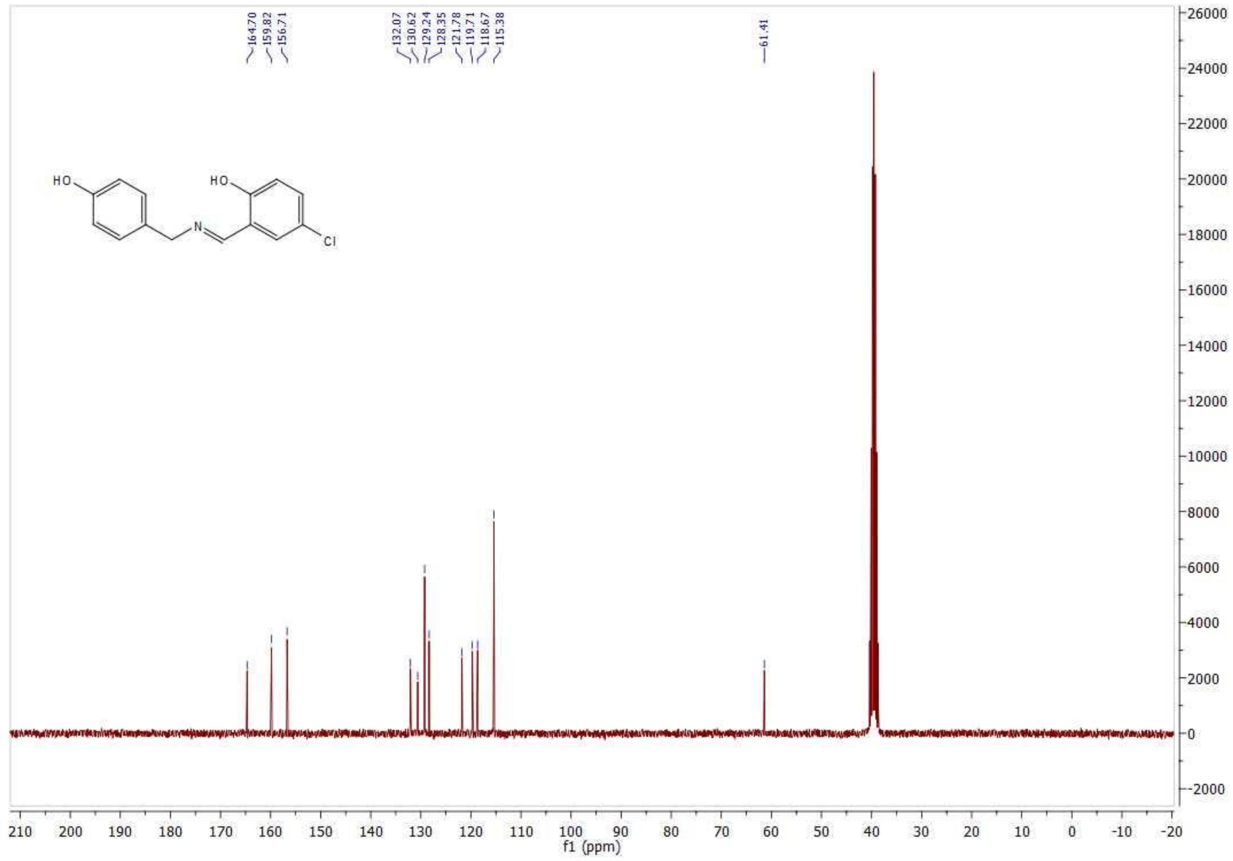

Figure S2. ^{13}C NMR spectra of compound **1**

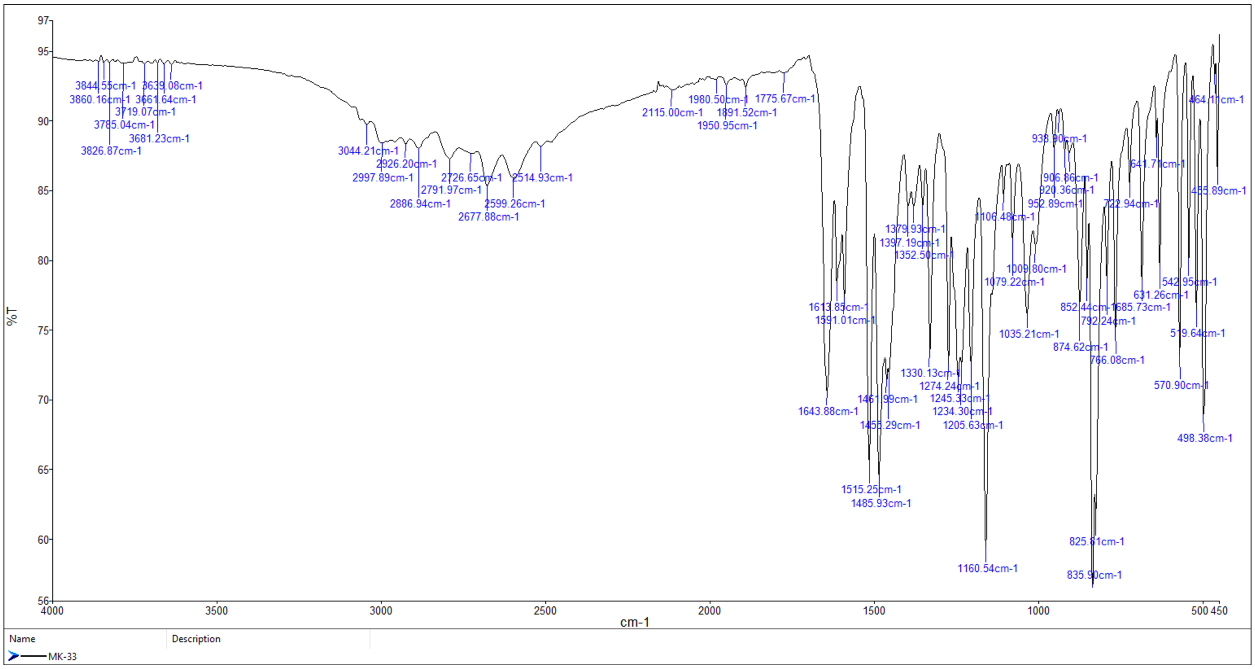

Figure S3. IR spectra of compound **1**

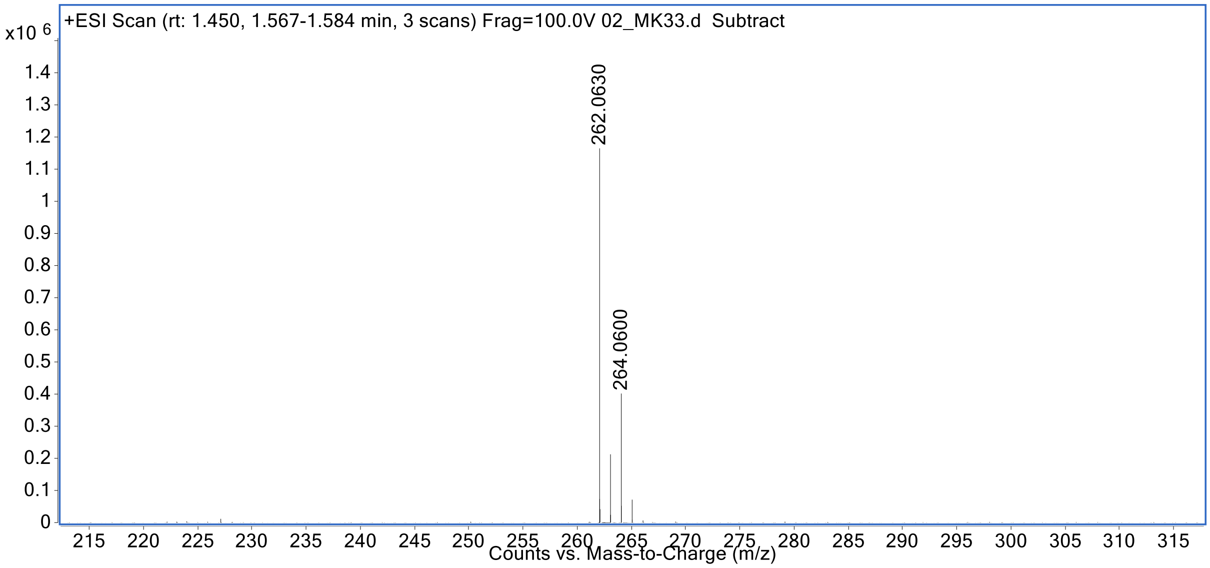

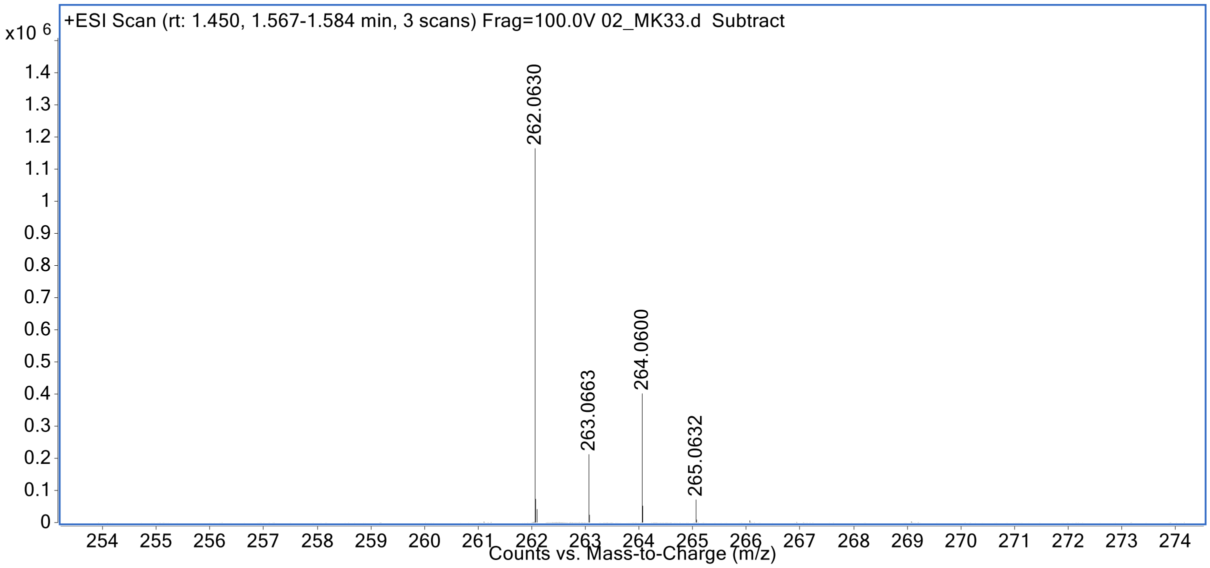

Figure S4. Mass spectrum of **1** in the m/z range 215–315 (upper spectrum) and its enlarged view in the m/z range 254–274 (lower spectrum).

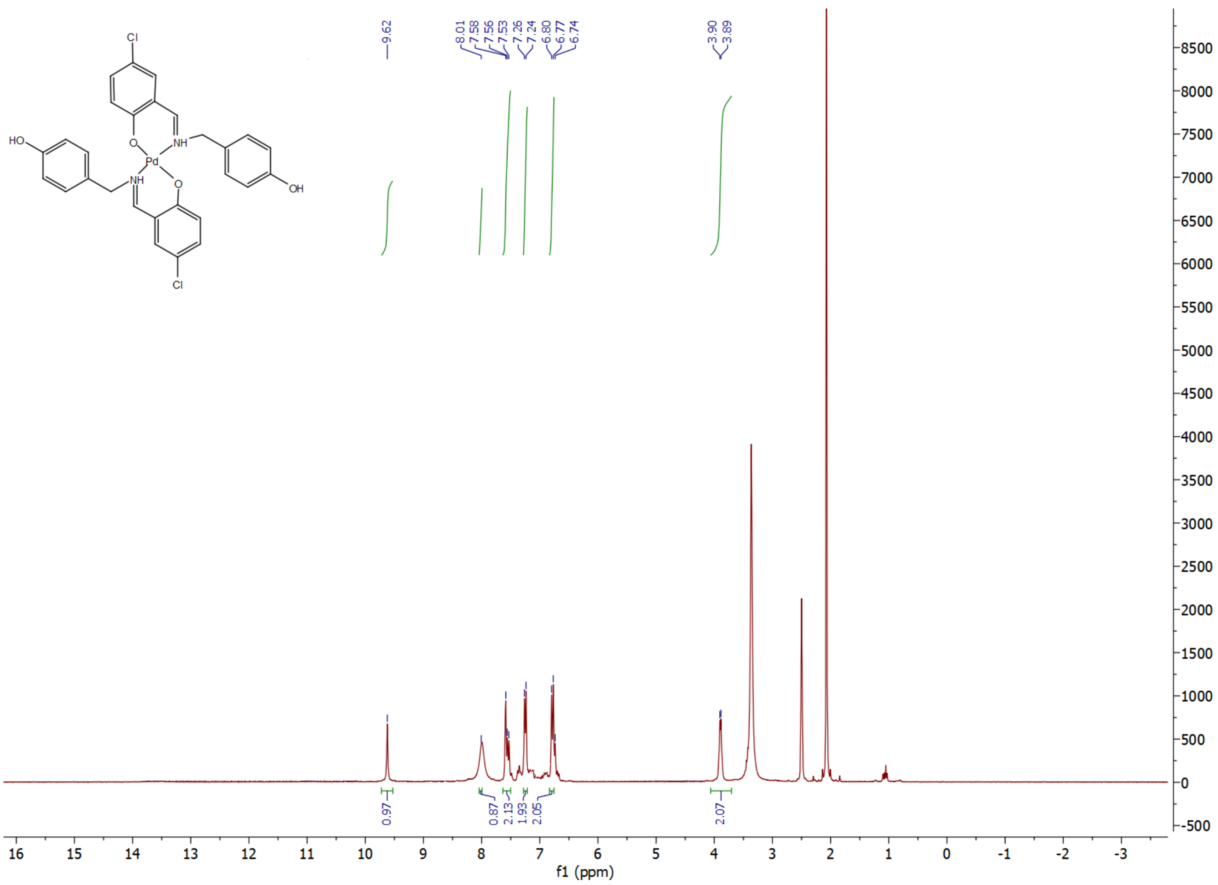

Figure S5. ^{1}H NMR spectra of compound **Pd1**

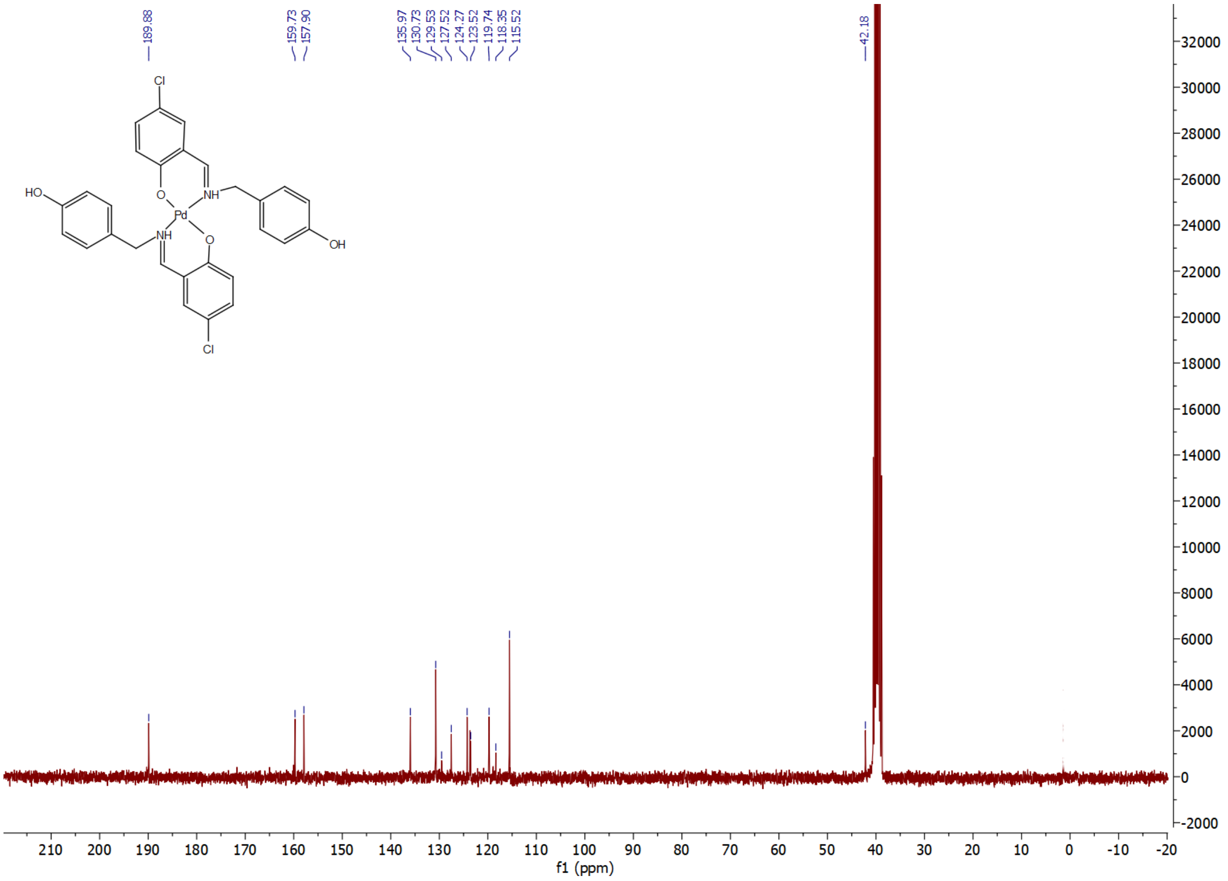

Figure S6. ^{13}C NMR spectra of compound **Pd1**

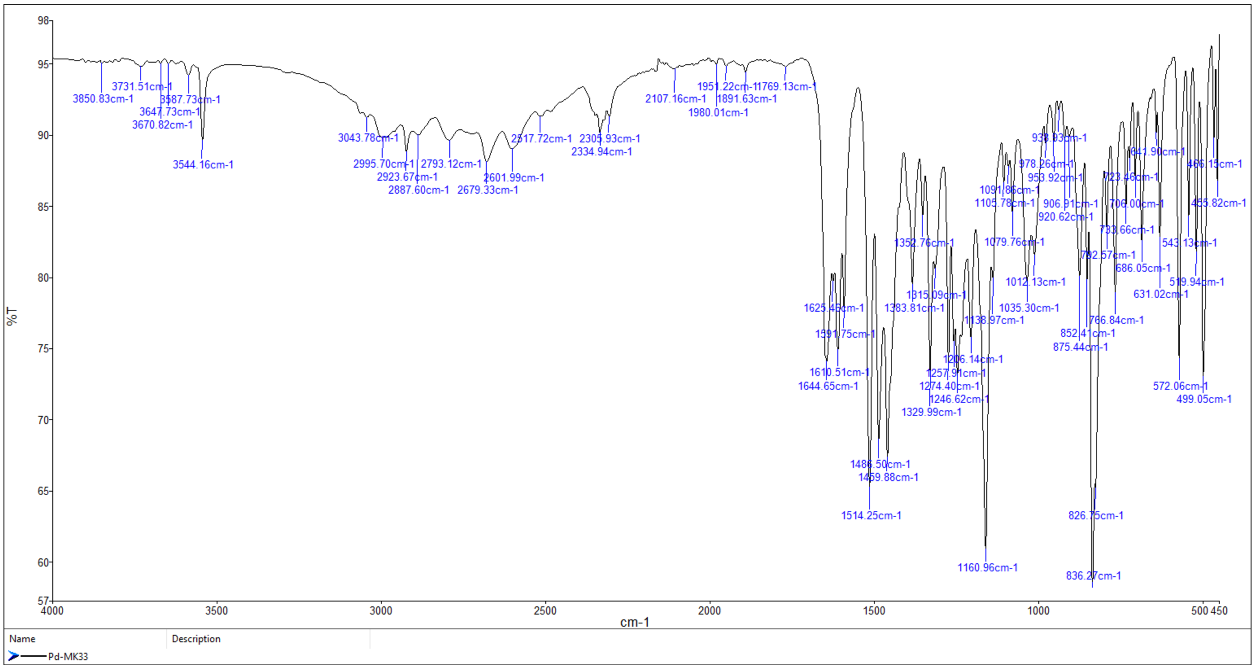

Figure S7. IR spectra of compound **Pd1**

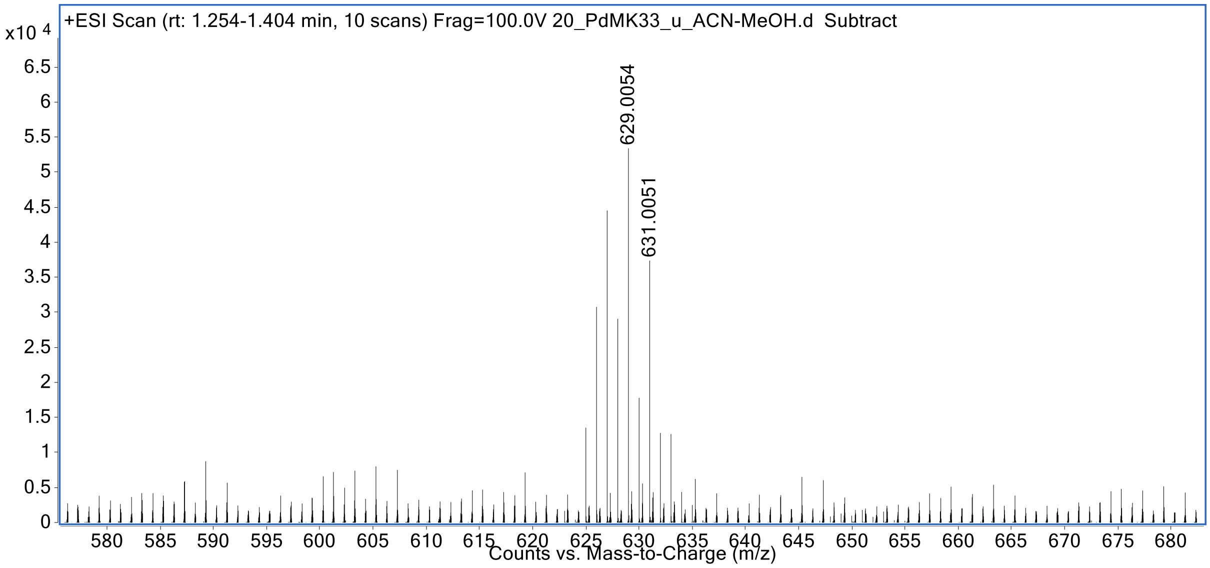

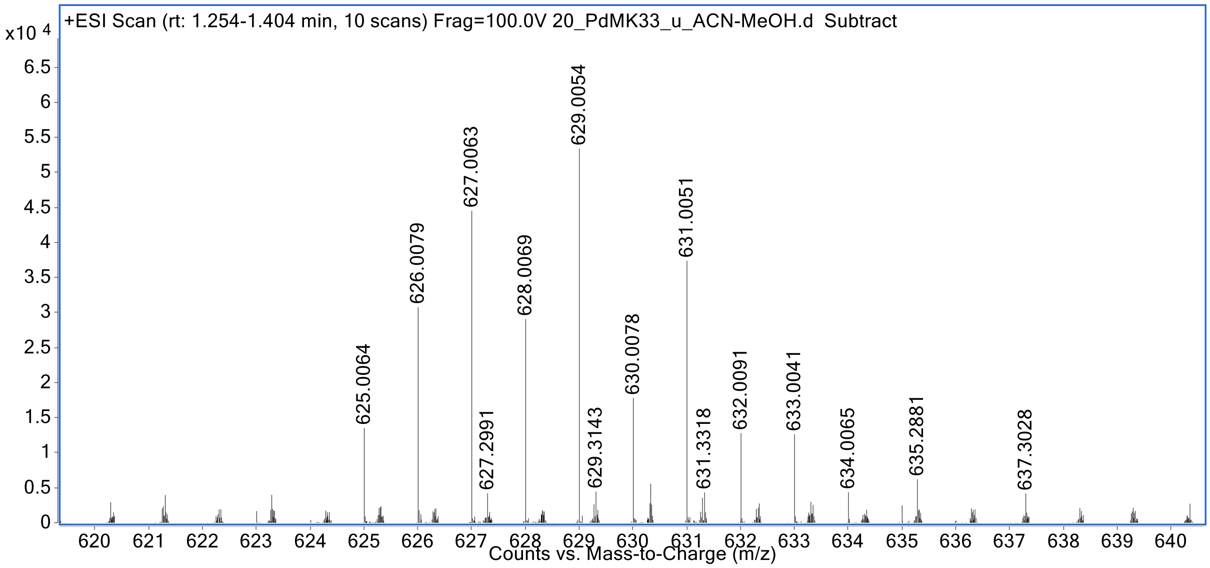

Figure S8. Mass spectrum of **Pd1** in the m/z range 215–315 (upper spectrum) and its enlarged view in the m/z range 254–274 (lower spectrum).

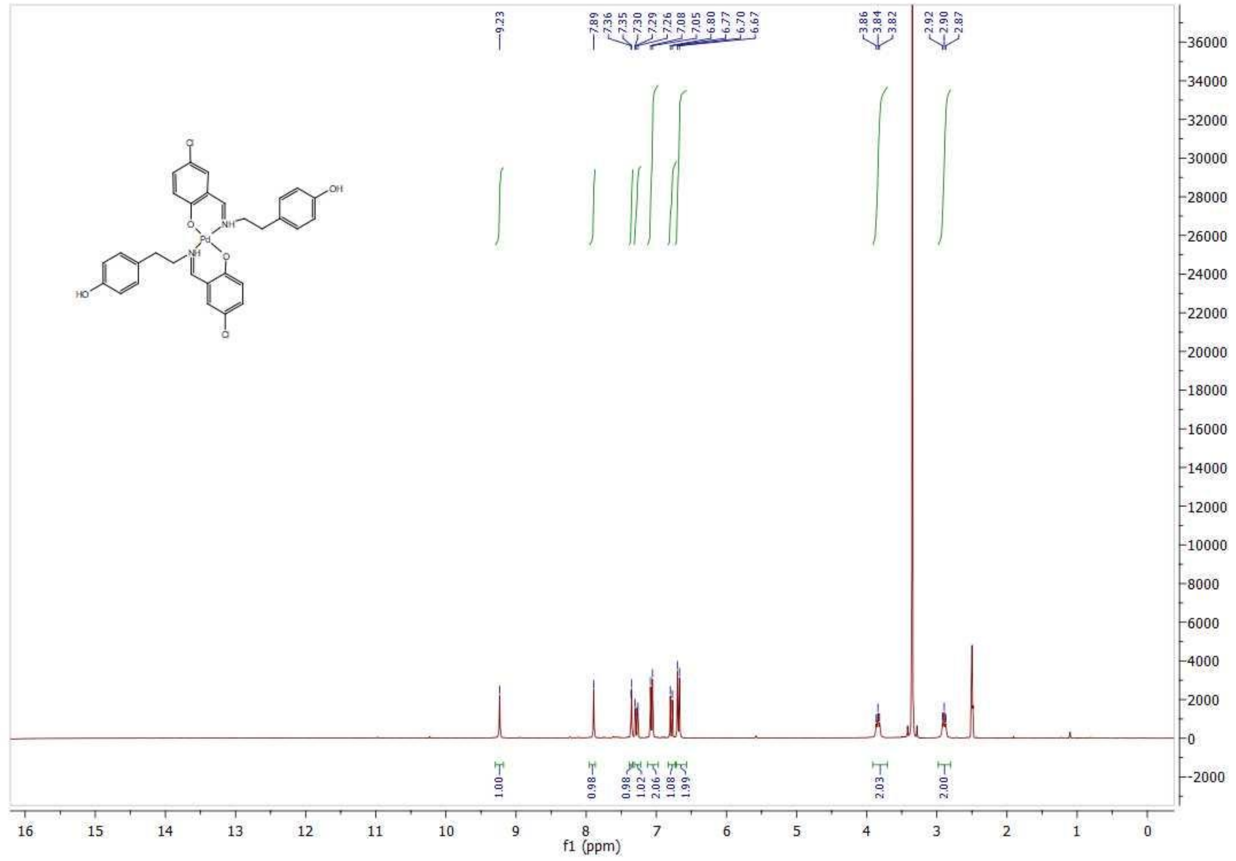

Figure S9. ^{1}H NMR spectra of complex **Pd2**

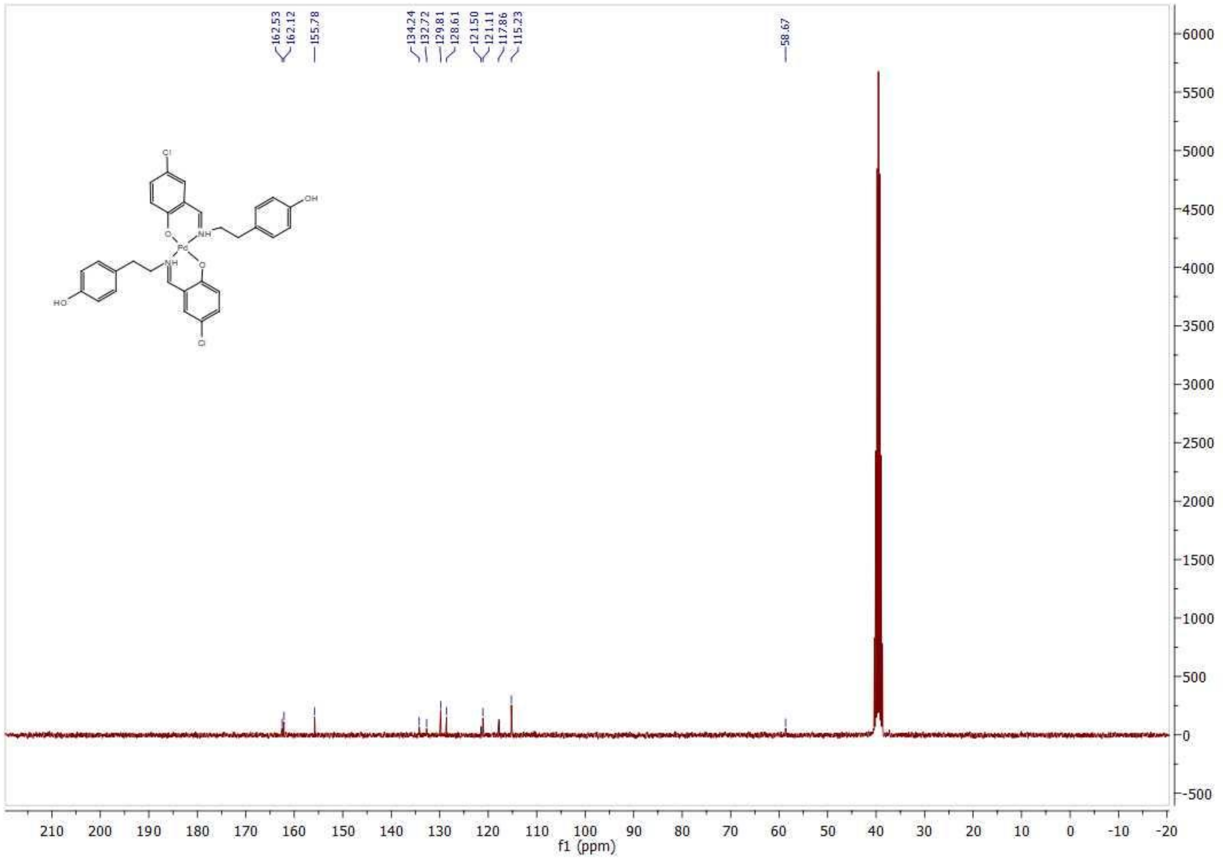

Figure S10. ^{13}C NMR spectra of complex **Pd2**

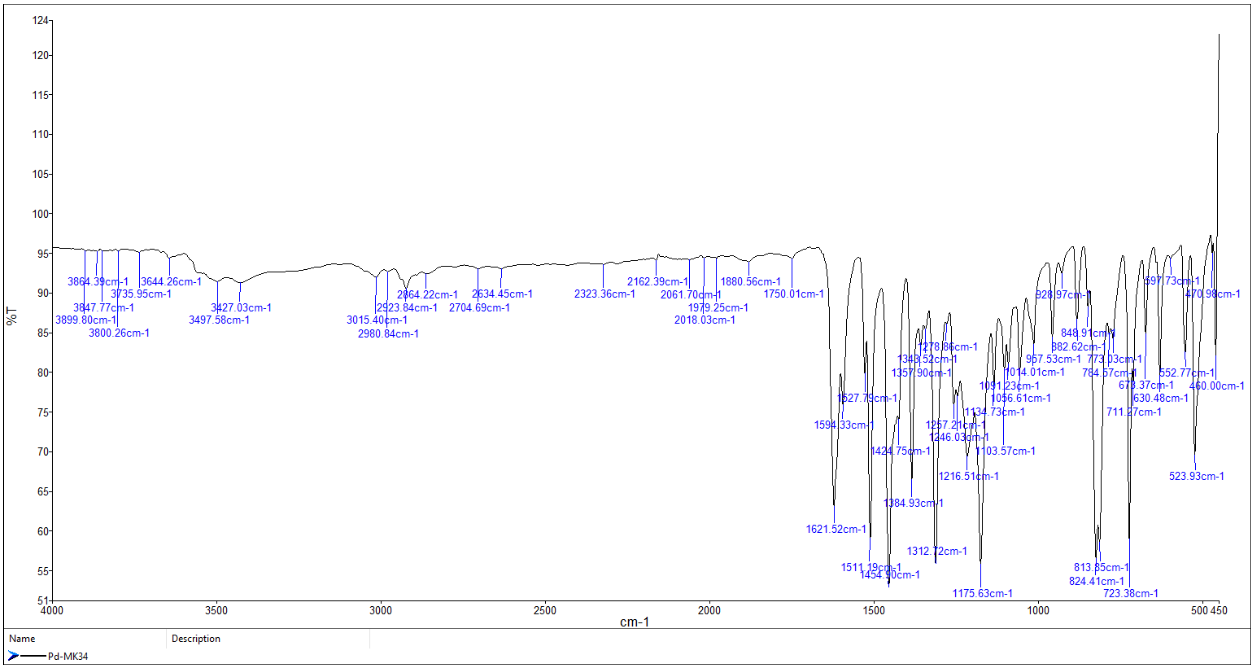

Figure S11. IR spectra of compound **Pd2**

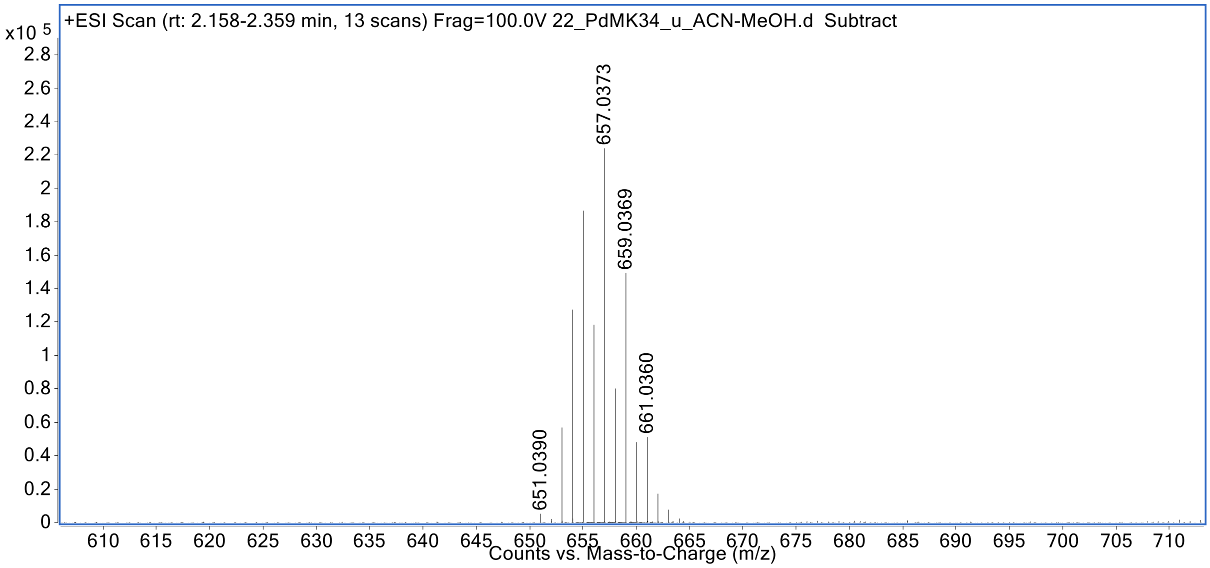

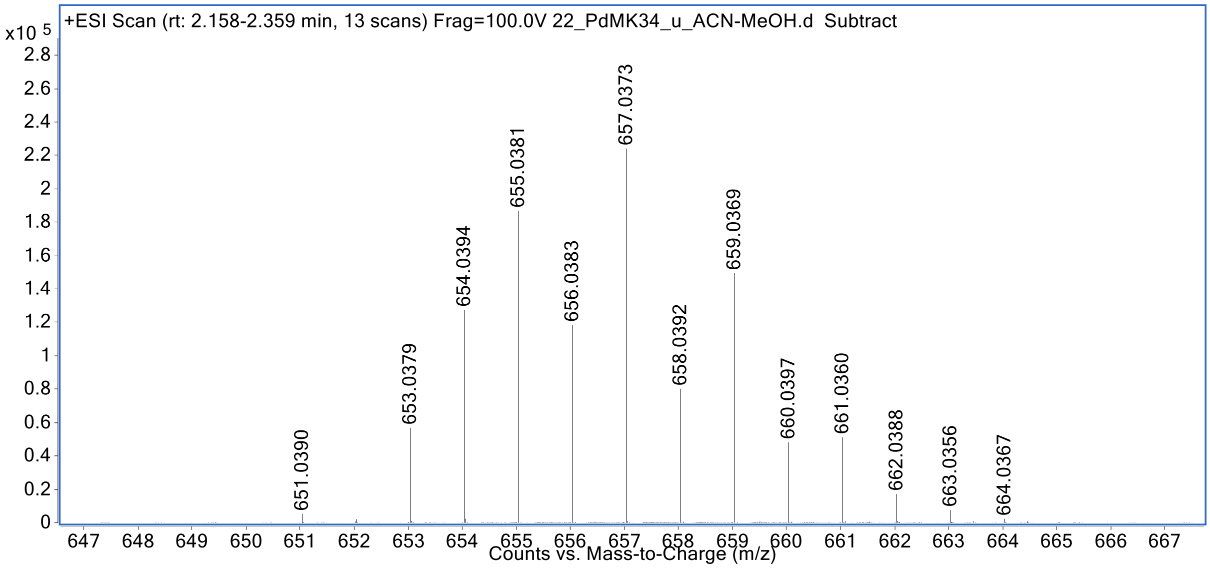

Figure S12. Mass spectrum of **Pd2** in the m/z range 215–315 (upper spectrum) and its enlarged view in the m/z range 254–274 (lower spectrum).

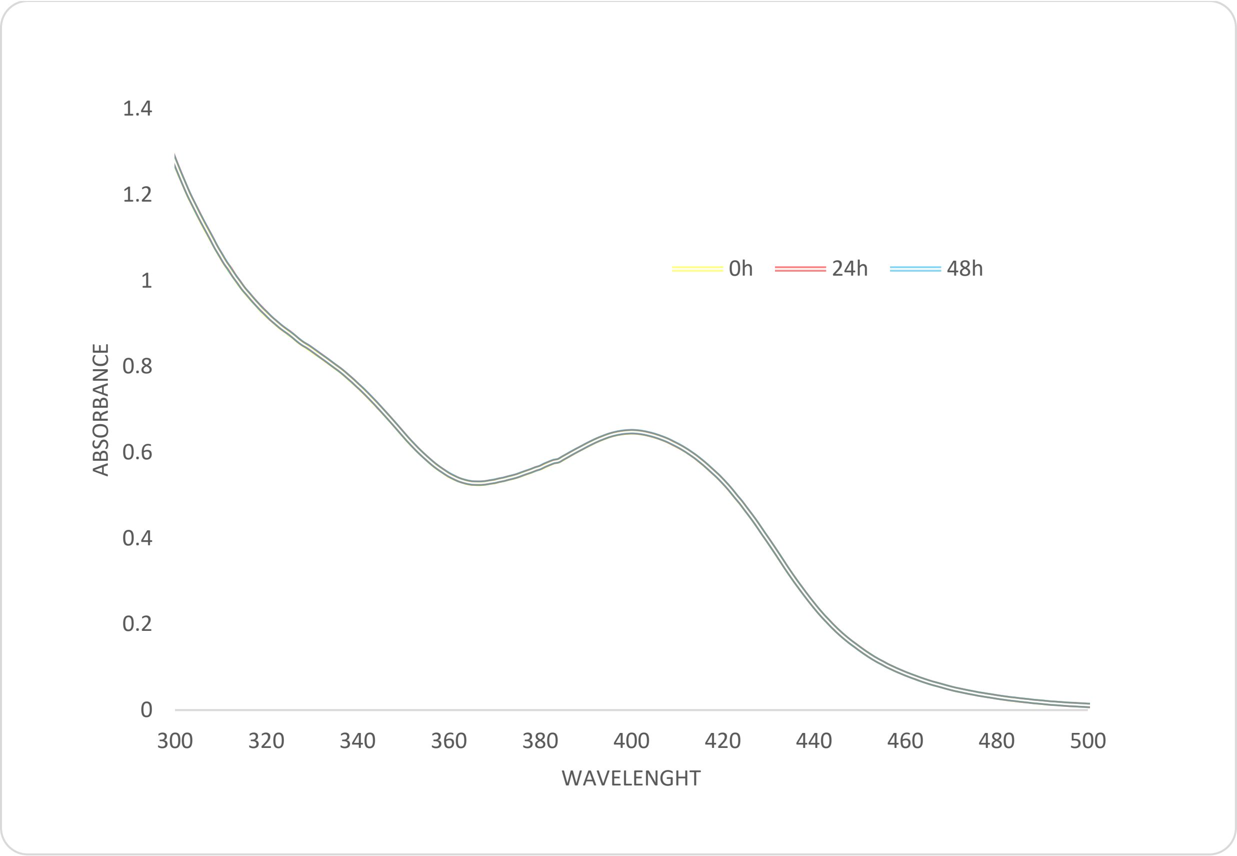

Figure S13. Stability assay of **Pd2** in PBS at 0, 48 and 72h

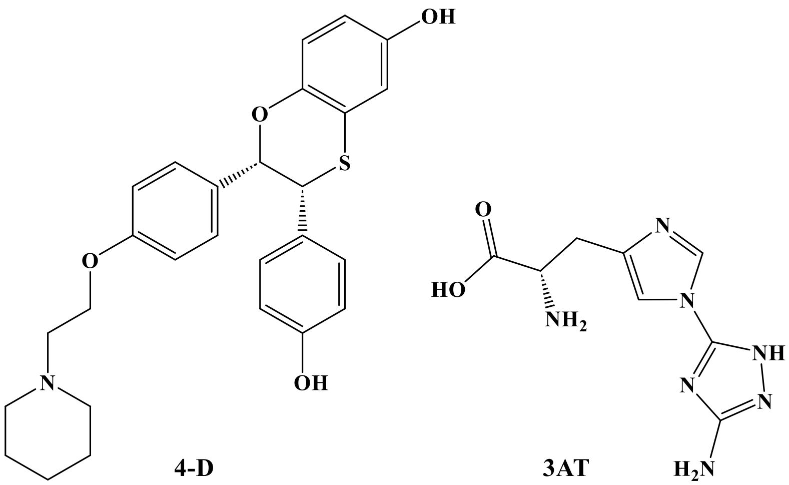

Figure S14. The structures of 4-D and 3AT
